# Host–parasite coevolution and the stability of genetic kin recognition

**DOI:** 10.1073/pnas.2220761120

**Published:** 2023-07-18

**Authors:** Thomas W. Scott, Alan Grafen, Stuart A. West

**Affiliations:** ^a^Department of Biology, University of Oxford, Oxford OX1 3SZ, United Kingdom

**Keywords:** evolution of altruism, kin discrimination, genetic kin recognition, host–parasite coevolution, Hamilton’s Rule

## Abstract

Crozier’s paradox suggests that genetic kin recognition will not be evolutionarily stable. The problem is that more common tags (markers) are more likely to be recognized and helped. This causes common tags to increase in frequency, eliminating the genetic variability that is required for genetic kin recognition. Two potential solutions to this problem have been suggested: host–parasite coevolution and multiple social encounters. We show that the host–parasite coevolution hypothesis does not work as commonly assumed. Host–parasite coevolution only stabilizes kin recognition at a parasite resistance locus if parasites adapt rapidly to hosts and cause intermediate or high levels of damage (virulence). Additionally, when kin recognition is stabilized at a parasite resistance locus, this can have an additional cost of making hosts more susceptible to parasites. However, we show that if the genetic architecture is allowed to evolve, meaning natural selection can choose the recognition locus, genetic kin recognition is more likely to be stable. The reason for this is that host–parasite coevolution can maintain tag diversity at another (neutral) locus by genetic hitchhiking, allowing that other locus to be used for genetic kin recognition. These results suggest a way that host–parasite coevolution can resolve Crozier’s paradox, without making hosts more susceptible to parasites. However, the opportunity for multiple social encounters may provide a more robust resolution of Crozier’s paradox.

## Significance

Kin selection theory predicts that individuals should evolve to help their relatives. However, Crozier’s paradox suggests that using genetic markers (tags) to recognize relatives will be evolutionarily unstable. The problem is that more common tags are more likely to be recognized and helped. This causes common tags to increase in frequency, eliminating the genetic variability needed for genetic kin recognition. It has been hypothesized that the genetic variability could be maintained if recognition alleles have an additional role in parasite resistance. We show that the host–parasite coevolution hypothesis does not work as expected because it often causes tags to fluctuate to high frequencies. However, host–parasite coevolution can maintain tag diversity at another (neutral) locus by genetic hitchhiking, resolving Crozier’s paradox.

Kin selection theory predicts that individuals should preferentially help their closer relatives (1, 2). The conditional helping of closer relatives, termed *kin discrimination*, is favored because relatives share genes, and so by helping a relative reproduce, an individual is still passing its genes to the next generation, just indirectly. This process requires *kin recognition*, which is the identification of relatives through either environmental or genetic cues (3–6).

**Fig. 1.**
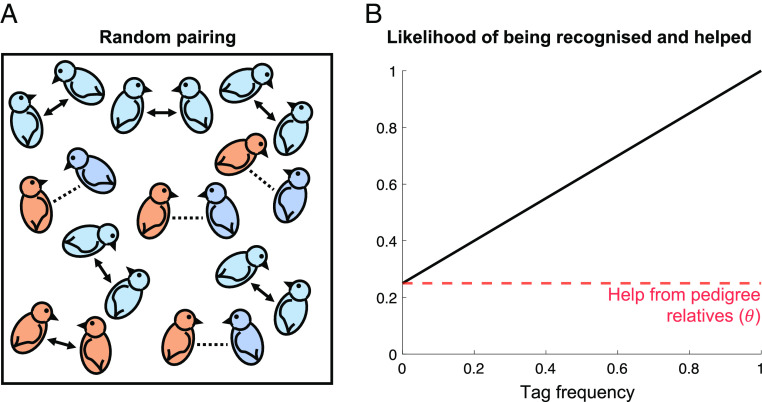
Crozier’s paradox. (*A*) Birds with a more common genetic tag (blue) are more likely to encounter birds with the same tag, compared to birds with a less common tag (orange). (*B*) Consequently, in the absence of multiple social encounters (*α*=0), individuals with more common tags are more likely to be recognized and helped (black solid line). Rare tags are only recognized and helped by pedigree (genealogical) relatives (red dotted line), but common tags are additionally recognized and helped by nonrelatives. We assumed parameter values that lead to Crozier’s paradox: *α*=0 (encounter parameter) *b*=0.4 (helping benefit), *c*=0.1 (helping cost), *µ_Trait_*=0 (trait mutation rate); individuals have a 25% chance of encountering a clone and a 75% chance of encountering a non-relative (*θ*=0.25; *θ* is population viscosity), meaning individuals evolve to help whoever they recognize (*θb > c*). Figure 1A previously published in ref. 22 and bird cartoons adapted from ref. 23, Creative Commons (https://creativecommons.org/licenses/by/3.0/).

Kin recognition via genetic cues is not necessarily evolutionarily stable (3, 5–13). The problem, known as Crozier’s paradox, is that more common tags (markers) at the recognition locus are more likely to be recognized (12). Consequently, individuals with more common tags are more likely to be helped, increasing their fitness (Fig. 1). In contrast, individuals with rare tags are less likely to be recognized and helped, reducing their relative fitness. This means that common tags will increase in frequency, and rare tags will decrease in frequency and be lost (positive frequency dependence). Therefore, genetic kin recognition drives its own ruin, by eliminating the genetic variability that is required for genetic kin recognition. Despite this potential for instability, genetic kin recognition has been observed in animals, microorganisms, and plants, suggesting that Crozier’s paradox has been solved by nature (4, 5, 14–21).

The most commonly accepted solution to Crozier’s paradox is that recognition alleles have an additional (pleiotropic) role unrelated to social behavior that maintains tag diversity (8, 9, 11, 12). Crozier was the first to suggest that natural selection would be favored to choose tag loci where there was already some other factor maintaining allele diversity (generating pleiotropy). Host–parasite coevolution could play this role of maintaining tag diversity because parasites evolve to infect common genotypes, and so rare host resistance alleles have an advantage (24–31). This hypothesis has been shown to work theoretically and has gained empirical support from cases where kin recognition appears to be based on tags that also have a function in host–parasite interactions—such as major histocompatability loci (MHC) in mammals, or the membrane of parasitic wasp larvae (8, 11, 32–37). Consequently, the host–parasite coevolution hypothesis had become the accepted explanation for stable genetic kin recognition.

However, there are at least three potential problems for the host–parasite coevolution hypothesis. First, we have recently shown that there is an alternative possible solution to Crozier’s paradox. If individuals can have multiple social encounters, then this would allow individuals with rare tags to find others with the same tag (22). This can prevent common tags from being more likely to be helped and hence eliminate Crozier’s paradox (22). In terms of Fig. 1*A*, this means that the orange birds find other orange birds to pair up with. Multiple social encounters are likely to be common in many cooperatively breeding birds and mammals, which can move around and choose who to help. It is not clear how such multiple encounters would interact with host–parasite coevolution. Would they act independently, synergistically, or even interfere?

Second, the empirical support for the host–parasite coevolution hypothesis has been questioned. The identification of the tags used to assess relatedness can be hard because they will usually correlate strongly with sharing across the rest of the genome. Most previous studies have not fully controlled for matching at other loci that could be used as tags and so while the data are consistent with MHC loci being used as tags, they are also consistent with many other possibilities (21). Green et al. (21) addressed this problem by developing a method to independently manipulate putative tags, while controlling genome-wide relatedness. They applied this method to house mice and found that genetic kin recognition was based on a urinary protein and not the MHC. Previous support for a role of the MHC could therefore just be an artifact of researchers preferentially examining MHC rather than an actual role for MHC.

Third, the theoretical basis of the host–parasite coevolution hypothesis remains unclear, even when acting alone. Host–parasite coevolution can lead to frequency fluctuations, with different alleles being favored at different times (24–31). These “Red Queen” fluctuations could reduce the usefulness of resistance alleles for tracking relatedness and even bring common tags closer to fixation, potentially destabilizing genetic kin recognition. Previous theory has assumed that rare alleles have a consistent advantage, rather than modeling the consequences of host–parasite coevolution (8, 11, 12). In addition, if an allele was used for both parasite resistance and genetic kin recognition, then this could lead to opposing selection pressures. For example, kin recognition could change tag frequency in a way that led to increased parasite susceptibility and reduced fitness. Host–parasite coevolution could therefore potentially reduce the likelihood that genetic kin recognition is favored.

We examine the theoretical plausibility of the host–parasite coevolution hypothesis, acting either alone or in combination with multiple social encounters. We develop a multi-locus population genetic model where resistance alleles are under a fluctuating selection pressure imposed by interactions with parasites, and multiple social encounters are possible. In addition, a key limitation of previous work is that it has assumed that genetic kin recognition must be based on a certain locus, such as that involved in parasite resistance. We allow the locus used for genetic kin recognition to evolve, so that natural selection can choose the recognition locus. By allowing the underlying genetic architecture controlling kin recognition to evolve, our model allows us to examine the consequences of interactions between different mechanisms and statistical associations (linkage disequilibria) between different possible recognition loci.

## Model

We constructed a theoretical population genetic model to examine when genetic kin recognition (tag diversity) can evolve to allow kin discrimination (conditional altruism). We give the full mathematical representation of our lifecycle assumptions in *SI Appendix*, *Appendix 1*. Briefly, we assumed an infinite population of initially haploid individuals, which interact in a population where relatives may reside near each other (viscous population). Each time an individual encounters a potential social partner, there is a probability *θ* of encountering a full clone (identical by descent at all loci) and a probability 1-*θ* of encountering a nonrelative (identical by descent at no loci). The parameter *θ* captures population viscosity (structure) and is therefore analogous to the “F-statistics” commonly used in population genetics to measure population structure/heterozygosity. Our decision to model population structure in this way, where probabilities of identity by descent are equivalent across all loci, is artificial but doesn’t appear to qualitatively affect results (*SI Appendix*, *Appendix 3*) (22).

### Recognition Loci.

Each individual has two candidate recognition alleles (tags), one segregating at a locus that has a role in parasite resistance (*Resist*) and one segregating at a locus that is neutral aside from its possible role in kin recognition (*Neutral*). The maximum number of tags that may simultaneously segregate at any one candidate recognition locus (genetic constraint) is given by *L_max_*. We allow the choice of recognition locus to evolve. Each individual has one of two possible alleles at a *Choice* locus, where one allele causes the individual to use *Resist* as a basis for genetic kin recognition, and the other allele causes the individual to use *Neutral*. Whenever an individual chooses *Resist* for kin recognition, its *Resist* allele becomes “pleiotropic” (influences more than one trait). Consequently, our model allows pleiotropy to evolve—this contrasts with previous models of kin recognition where pleiotropy was assumed (9, 11, 12).

### Social Encounters.

**Fig. 2.**
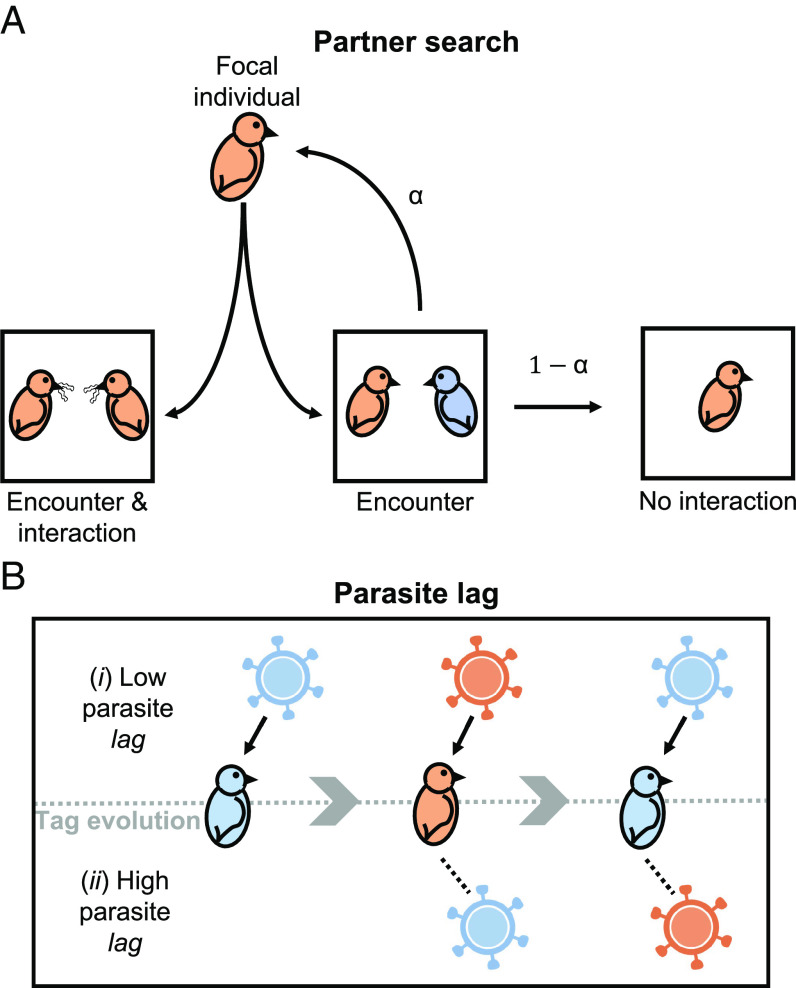
Lifecycle features. (*A*) Social encounters and social interactions. If the focal individual encounters a tag-matched individual (both orange), it socially interacts. Conversely, if the focal individual encounters a tag-mismatched individual (one orange; one blue), the focal individual may encounter a new partner (α), or forgo the social search (1-α). Higher values of the encounter parameter (α) correspond to individuals having more encounters to find a matching partner (partner search). During an interaction with a (tag-matched) partner, the focal individual may help or not (defect), depending upon its allele at the trait locus. (*B*) Host–parasite coevolution. As tag frequencies change (gray arrows), parasites may (i) adapt rapidly (low *lag*), meaning the most common tag faces the highest probability of infection (birds and viruses match); (ii) adapt slowly (high *lag*), meaning the most common tag may not face the highest probability of infection (birds and viruses don’t match). If infected by a parasite, an individual’s fecundity is reduced by *d*. Figure 2A previously published in ref. 22 and bird cartoons adapted from ref. 23, Creative Commons (https://creativecommons.org/licenses/by/3.0/).

Each individual encounters someone in the viscous population and has an opportunity to provide help (Fig. 2*A*). If an individual, at its chosen recognition locus (either *Resist* or *Neutral*), shares a tag with its partner, it interacts and potentially helps—the social encounter becomes a social interaction. In contrast, if an individual doesn’t share a tag with its partner, what happens depends upon the encounter parameter, α (22). With a probability α, an individual with a tag-mismatched partner will abandon that partner and reassociate for a new social encounter, with someone new in the viscous population. With a probability 1-α, an individual with a tag-mismatched partner remains with that partner, but it does not interact (the opportunity to provide help is wasted) (Fig. 2*A*). The parameter α can be thought of as capturing the potential effort that individuals are willing to put into partner search (22). Mathematically, the generational probability that a given individual finds a tag-matched partner to interact with and potentially help is $\frac{\theta +\left(1-\theta \right)X}{1-\alpha \left(1-X\right)\left(1-\theta \right)}$ , where *X* is the population frequency of the individual’s tag (allele at its chosen recognition locus) and *θ* is population viscosity (structure).

### Social Interactions.

We assumed that, when an individual successfully finds (encounters) a partner with the same tag at its chosen recognition locus, it interacts and potentially helps. Whether an individual helps depends upon its allele at the helping (*Trait*) locus. Individuals with a “conditional helping” allele will help, paying a fecundity cost of *c* to give a benefit of *b* to their social partner. Individuals with a “defect” allele do not help.

### Host–Parasite Coevolution.

In each generation, each individual may be infected by a parasite. The probability of being infected depends on which parasite resistance allele it has at the *Resist* locus. We assumed that parasites evolve to exploit the parasite resistance allele that is most common in the population, but that this can take time (generations). The result is that an individual’s probability of being infected by a parasite is not necessarily given by the current population frequency of its parasite resistance allele (*Resist*) (Fig. 2*B*). Rather, it is given by some previous population frequency of its parasite resistance allele, recorded *lag* generations ago (38). The parameter *lag* corresponds to the amount of evolutionary time that the parasite population needs to adapt to the most common parasite resistance allele, with *lag*=0 implying perfect parasite adaptation each host generation (successful exploitation of the currently most common parasite resistance allele). Previous host–parasite models have shown that the rate at which parasites adapt to their hosts can depend upon a number of factors, such as relative generation time (28–30, 39, 40). If infected, an individual’s fecundity is reduced by *d*, where the parameter *d* corresponds to parasite virulence. Mathematically, a given individual will suffer a generational parasite-induced fecundity loss of dZ , where Z is the population frequency of the individual’s *Resist* allele, recorded *lag* generations ago.

We modeled parasites in this abstract way, rather than letting them evolve, because it allowed us to directly adjust the key high-level factors (rate of adaptation and virulence), rather than give a specific account of how these high-level factors arise from more fundamental variables such as relative generation time, population sizes, and the probability of parasite coinfection. Direct manipulation of the key parameters gives us a clearer causal understanding of the evolutionary forces at play than letting these high-level factors emerge from the complex and context-specific interactions of lower-level variables (41–43).

After social interactions and host–parasite interactions have taken place, haploid individuals produce a very large number of gametes before dying, where an individual’s fecundity is given by the product of its payoffs from social and host–parasite interactions. This is followed by gametes fusing randomly, then meiosis with free recombination occurring between each of the four loci (no physical linkage), and then mutation at the *Trait* and *Choice* loci occurring with respective probabilities *µ_Trait_* and *µ_Choice_*. Finally, a number of haploid adults are sampled randomly from the haploid juvenile population, such that the adult population remains constant in size over generations. In *SI Appendix*, *Appendix 1*, we give explicit equations for: individual fitness (*SI Appendix*, Eqs. **S1–S3**); recombination-induced genotype frequency change (*SI Appendix*, Eq. **S4**); and mutation-induced genotype frequency change (*SI Appendix*, Eq. **S5**). Taken together, these equations describe how genotype frequencies change across any given generation. We also give precise mathematical definitions for tag diversity (*SI Appendix*, Eqs. **S6 and S7**) and parasite susceptibility (*SI Appendix*, Eq. **S10**), which we track and record at equilibrium, alongside *Trait* and *Choice* allele frequencies.

## Results

### Host–Parasite Coevolution and Genetic Kin Recognition.

Our first aim was to examine whether host–parasite coevolution alone can facilitate genetic kin recognition. We considered the special case where all individuals have the *Choice* allele that makes them base genetic kin recognition on the parasite resistance locus (*Resist*); the choice of recognition locus cannot evolve (*µ_Choice_*=0); and individuals cannot have multiple social encounters (α=0).

### Can Host–Parasite Coevolution Stabilize Genetic Kin Recognition at the Parasite Resistance Locus?

In *SI Appendix*, *Appendix 2*, we show that genetic kin recognition is stable if two conditions are met. The first condition for genetic kin recognition to be stable is that kin discrimination must be favored by kin selection. By this, we mean that conditional helping (help if matching tag) must have a higher inclusive fitness payoff than both defection (never help) and indiscriminate helping (always help, irrespective of tag). This occurs when Hamilton’s rule is met, giving *R_tag_ b* > *c*, where *R_tag_* is the relatedness between actors and their (tag-matched) social interactants (1, 2, 4, 22, 44–53). Here, relatedness technically means genetic similarity at the *Trait* locus, but at evolutionary equilibrium, this will usually be equal to the probability that individuals share common ancestry (pedigree/genealogical relatedness; e.g., 1/2 for full siblings, 1/8 for cousins) (*SI Appendix*, *Appendix 8*) (1, 2, 22, 49, 54). This condition arises because, if it is not satisfied, helping is not favored, meaning discriminatory help (genetic kin recognition) cannot evolve at equilibrium. In *SI Appendix*, *Appendix 2*, we derive relatedness as

[1] $$\begin{array}{c} R_{\mathrm{tag}}=\frac{\frac{\theta +\left(1-\theta \right)Xp}{\theta +\left(1-\theta \right)X}-\bar{p}}{1-\bar{p}},\; \end{array}$$

where θ is population viscosity; *X* is the population frequency of the actor’s tag (chosen recognition allele); *p* is the proportion of individuals with the actor’s tag that are helpers; and $\bar{p}$ is the proportion of the population who are helpers.

**Fig. 3.**
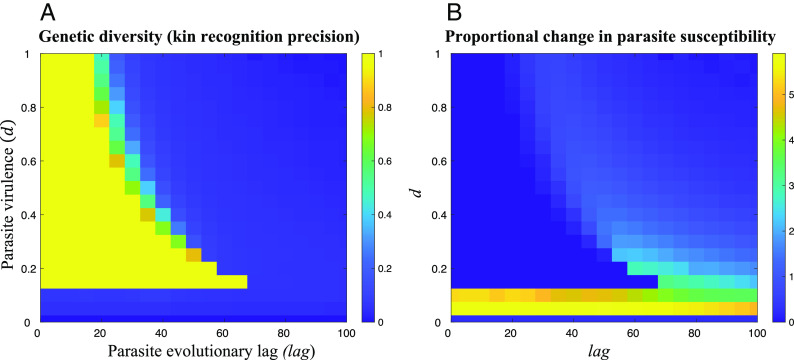
Genetic kin recognition and parasite susceptibility when a parasite resistance locus is used for recognition. We plot (*A*) the equilibrium genetic diversity at a parasite resistance locus that is also being used for kin recognition (*Resist*); (*B*) the extent to which using *Resist* for kin recognition leads to an increased susceptibility to parasites, relative to when not using *Resist* for kin recognition. Genetic diversity is scaled between 0 and 1 (*SI Appendix*, Eq. **S7**), and parasite susceptibility is the equilibrium average generational probability of being infected by a parasite (*SI Appendix*, Eq. **S10**). We assumed: *θ*=0.25 (population viscosity), *b*=0.3 (benefit of helping), *c*=0.1 (cost of helping), *L_max_*=10 (maximum number of tags); α=0 (encounter parameter); *μ_Trait_*=0.001 (*Trait* mutation rate); such that kin discrimination is favored by kin selection (*R_tag_ b* – *c* > 0), meaning the conditional helping allele is positively selected whenever appreciable tag diversity is maintained.

The second condition for genetic kin recognition to be stable is that rare tags must be maintained in the population, so that there is sufficient genetic diversity at the recognition locus (*Neutral*) to allow genetic kin recognition. By iterating our genotype frequency recursions (*SI Appendix*, Eqs. **S1–S5**) in the area of parameter space where *R_tag_ b* > *c* is satisfied, we found that genetic kin recognition is only stable if parasites evolve rapidly to better infect common genotypes (low *lag*) and have intermediate or (provided that *lag* is *very* low or zero) high virulence (*d*) (Fig. 3*A*).

Rapid parasite evolution (low *lag*) facilitates tag diversity because, if parasites evolve rapidly to the currently most common tag in the population (low *lag*), this means that rare tags are more likely to have an advantage over common tags in any given generation. Conversely, slow parasite evolution (high *lag*) means that, in any given generation, parasites are less likely to be targeting the most common tags—instead, they will be targeting tags that were common in some previous generation, but which may not be common anymore. This means that common tags are more likely to run away to fixation, eliminating tag diversity. Put simply, if host–parasite coevolution is too slow, then it cannot prevent kin recognition from eliminating tag diversity.

Intermediate virulence (*d*) facilitates tag diversity because, if *d* is too low, the force of extrinsic balancing selection is too weak to overturn Crozier’s paradox and stabilize tag diversity. Conversely, if *d* is too high, tags are more likely to cycle severely in frequency, because any tags that are selected in one generation are likely to shoot rapidly up in frequency. The tag frequency oscillations associated with high parasite virulence (*d*) serve to destabilize tag diversity. However, if parasites adapt very rapidly or instantaneously, tag diversity can be stabilized even for very high parasite virulence (setting *lag* to be very low or zero removes the upper limit on *d*). This agrees with Rousset & Roze’s implicit analysis of the *lag*=0 case (11).

**Result 1:** *Host–parasite coevolution can facilitate genetic kin recognition at the parasite resistance locus, but this requires that parasites are rapidly adapting with intermediate or high virulence.*

### Does Genetic Kin Recognition at the Parasite Resistance Locus Lead to Increased Parasite Susceptibility?

In addition, in *SI Appendix*, *Appendix 3*, we show that using a parasite resistance locus to stabilize kin recognition can increase parasite susceptibility (Fig. 3*B*) (55). Technically, we equate parasite susceptibility with the generational probability of being infected by a parasite, which in turn is given by the frequency, recorded *lag* generations ago, of the individual’s *Resist* allele (*SI Appendix*, Eq. **S10**). The reason for this cost of kin recognition is that, in the region of parameter space where host–parasite coevolution facilitates kin recognition, social interactions will favor common recognition alleles over rare recognition alleles. This can reduce diversity at the resistance locus, increasing parasite susceptibility, and reducing fitness. The only situation in which this does not occur in our model is when host–parasite coevolution provides very strong extrinsic balancing selection (e.g., very low or zero *lag*; high *d*), allowing diversity at the resistance locus to be approximately maximized, so that the increase in parasite susceptibility is negligible.

**Result 2:** *Using a parasite resistance locus for kin recognition can lead to increased parasite susceptibility*

### The Joint Influence of Host–Parasite Coevolution and Multiple Social Encounters on Genetic Kin Recognition.

We then examined the consequences of allowing for both host–parasite coevolution and multiple social encounters at the same time, to determine how these mechanisms interact. In addition, we assumed that both *Choice* alleles are initially in the population, so that genetic kin recognition could be based on either the parasite resistance locus (*Resist*) or the otherwise-neutral locus (*Neutral*). This assumption allows the locus used for genetic kin recognition to evolve, so that natural selection can choose the recognition locus. We are therefore examining the consequences of allowing the genetic architecture underlying kin recognition to evolve.

### At What Locus is Recognition Favored?

We show in *SI Appendix*, *Appendix 4* through iteration of our genotype frequency recursions that the population evolves in the direction of using the candidate recognition locus with the most tag diversity at that point in time (*SI Appendix*, Eqs. **S1–S5**). In other words, there is directional selection at the *Choice* locus, with the positively selected *Choice* allele being the one that chooses the candidate kin recognition locus with the most tag diversity. The result is that, once tag diversity has equilibrated at each candidate recognition locus (evolutionary long term), individuals use the candidate recognition locus that maintains the most tag diversity. If equal tag diversity is maintained at each candidate recognition locus, then individuals may use either candidate recognition locus without selective consequence (no long-term *Choice* selection).

**Result 3:** *Individuals evolve to use the recognition locus that maintains the most tag diversity*

**Fig. 4.**
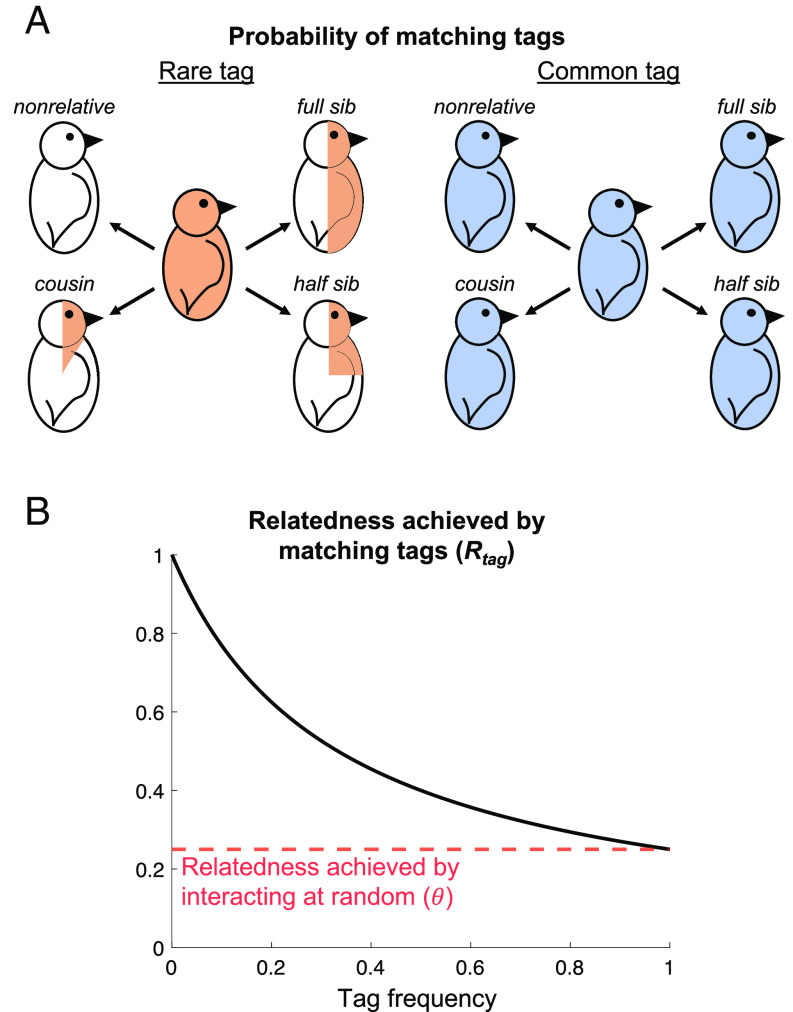
Relatedness decreases with tag frequency. (*A*) For birds with rare tags (orange), the probability of matching tags with someone (proportion shaded) is given by pedigree relatedness. For birds with common tags (blue), the probability of matching tags with someone (proportion shaded) is always high, regardless of pedigree relatedness. More common tags are therefore worse indicators of both relatedness at the trait locus and pedigree relatedness. (*B*) Therefore, the relatedness achieved by matching tags (black solid line) decreases with tag frequency. A tag at fixation achieves the same relatedness as choosing social partners at random (no tag used; red dashed line). We assumed: $\overset{-}{p}=p_{i}$ (no tag-trait linkage disequilibrium); θ=0.25 (population viscosity). The full *R_tag_* expression is given in Eq. **1**. Figure 4A previously published in ref. 22 and bird cartoons adapted from ref. 23, Creative Commons (https://creativecommons.org/licenses/by/3.0/).

The locus with the most tag diversity is favored because it allows more precise kin recognition (Fig. 4). For loci with high tag diversity, each tag will have a low population frequency, meaning different families are likely to have different tags. Tag-matching at this locus is therefore more likely to identify members of your family (pedigree relatives), resulting in precise kin recognition. In contrast, for loci with low tag diversity, each tag will have a relatively high population frequency, meaning different families are likely to share tags. Tag-matching at this locus is at risk of erroneously identifying pedigree nonrelatives instead of relatives, resulting in imprecise kin recognition (help given to nonrelatives). This relationship between tag diversity and relatedness can be seen by the following: first, noting that a high tag diversity implies that each tag has a low population frequency (*SI Appendix*, Eq. **S6/S7**); then, inspecting Eq. **1**, to see that a low population frequency (*X*) is associated with a high value of *R_tag_* (more precise kin recognition).

**Fig. 5.**
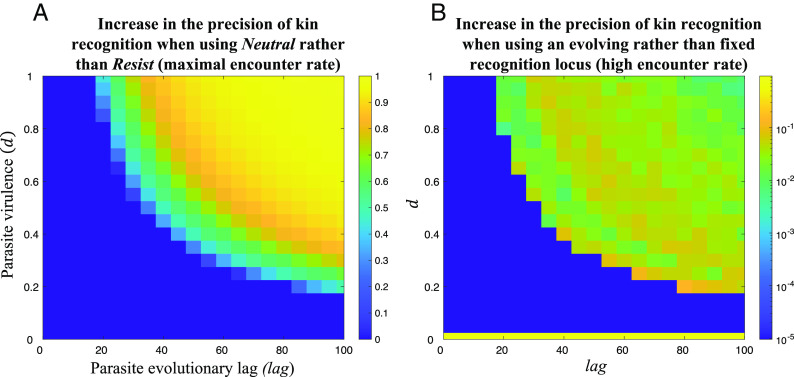
Genetic kin recognition when there are multiple social encounters and natural selection can choose the recognition locus. (*A*) We plot on a linear scale: the equilibrium genetic diversity (kin recognition precision) at an otherwise-neutral locus (*Neutral*), minus the equilibrium genetic diversity at a parasite resistance locus (*Resist*), when social encounters are unlimited (α=1). Host–parasite coevolution can oppose multiple social encounters and reduce tag diversity. (*B*) We plot on a log scale: the maximum equilibrium genetic diversity that arises at the recognition locus when natural selection chooses the recognition locus, minus the maximum equilibrium genetic diversity that can arise when the recognition locus is fixed at either *Neutral* or *Resist*, when the social encounter parameter is high but not maximal (α=0.99). Letting the recognition locus evolve increases tag diversity at the recognition locus. We assumed: *θ*=0.25 (population viscosity), *b*=0.3 (benefit of helping), *c*=0.1 (cost of helping), *L_max_*=10 (maximum number of tags), *μ_Trait_*=0.001 (*Trait* mutation rate), *μ_Choice_*=0.001 (*Choice* mutation rate); such that kin discrimination is favored by kin selection (*R_tag_ b* – *c* > 0), meaning the conditional helping allele is positively selected whenever appreciable tag diversity is maintained.

When kin recognition can be based on either recognition locus, different mechanisms may be responsible for initially maintaining tag diversity at each locus. First, host–parasite coevolution can maintain diversity at the resistance locus (*Resist*), but only if parasites adapt sufficiently quickly, as discussed earlier (Fig. 5*A*; Result 1). Second, as we have shown both here (*SI Appendix*, *Appendix 3, Scenario 1* and *Appendix 4*) and in a previous study (22), multiple encounters (high α) can maintain diversity at the otherwise-neutral locus (*Neutral*). Multiple social encounters remove the benefit of having a common tag (Fig. 1) and so prevent genetic variability from being eliminated at the otherwise-neutral locus (*Neutral*) (22). This then gives time for helping and kin recognition to coevolve, allowing *Neutral* tag diversity to actively build up, in the following way (22, 54, 56). As tags become more common, they become less useful cues of pedigree relatedness (common ancestry), meaning kin selection is less likely to favor the helping of tag-matched individuals (this can be seen by noting in Eq. **1** that *R_tag_* decreases with tag frequency, *X*). Consequently, defection can invade at common tags. In contrast, rare tags are good indicators of relatedness, meaning kin selection favors the helping of tag-matched individuals. This means that rare tags cannot be invaded by defectors. Technically, a statistical association between genes for helping and rare tags builds up (the statistical association is mathematically defined in *SI Appendix*, Eq. **S21** and tracked in *SI Appendix*, *Appendix 5*) (22). The consequence of this coevolution is that individuals with rare tags have a greater average payoff from social interactions, meaning rare tags increase in frequency, maintaining tag diversity (22, 54, 56). We note that a small amount of *Trait* mutation (*µ_Trait_*) is sometimes required for multiple social encounters to stabilize neutral tag diversity, as explained in *SI Appendix*, *Appendix 6* (22).

### How Does the Evolution of Tag Locus Influence the Stability of Genetic Kin Recognition?

We then examined how the evolution of the choice of recognition locus influenced tag diversity. We show in *SI Appendix*, *Appendix 4* that, when the choice of recognition locus is allowed to evolve, tag diversity can build up over a greater region of parameter space than when the recognition locus is fixed at either the parasite resistance (*Resist*) or otherwise-neutral (*Neutral*) locus (Fig. 5*B*).

**Result 4:** *Letting the choice of tag locus evolve increases the parameter space where genetic kin recognition is stable.*

**Fig. 6.**
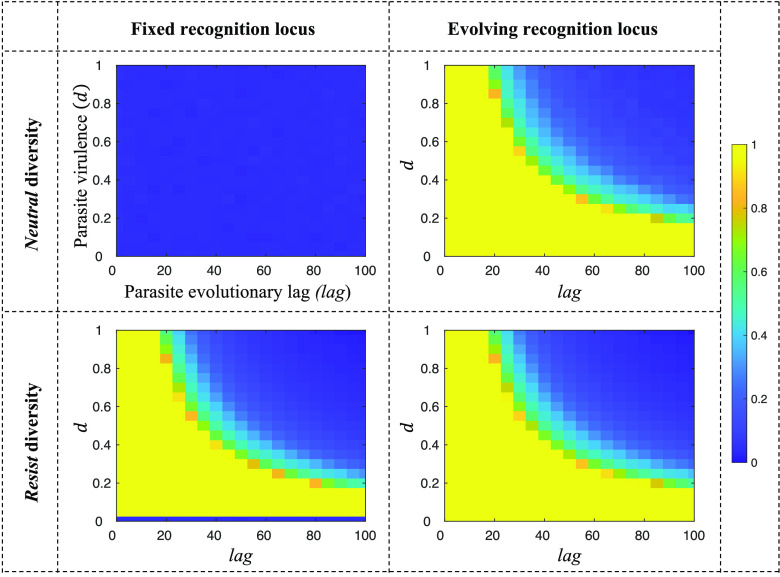
Host–parasite coevolution can cause diversity to build up at an otherwise-neutral recognition locus by genetic hitchhiking. We plot the equilibrium genetic diversity at a parasite resistance locus (*Resist*), and an otherwise neutral locus (*Neutral*), when the loci are forced to be used for kin recognition (fixed recognition locus; *Choice* mutation: *μ_Choice_*=0) and when natural selection can choose the recognition locus (evolving recognition locus; *Choice* mutation: *μ_Choice_*=0.001). Social encounters are assumed to be high but not unlimited (intermediate; α=0.99). *Neutral* tag diversity only builds up when the recognition locus can evolve, by genetic hitchhiking. We assumed: *θ*=0.25 (population viscosity), *b*=0.3 (benefit of helping), *c*=0.1 (cost of helping), *L_max_*=10 (maximum number of tags), *μ_Trait_*=0.001 (*Trait* mutation rate); such that kin discrimination is favored by kin selection (*R_tag_ b* – *c* > 0), meaning the conditional helping allele is positively selected whenever appreciable tag diversity is maintained.

The evolution of the tag locus increases the area where genetic kin recognition is stable because it allows *genetic hitchhiking* (57–59) (Fig. 6 and *SI Appendix*, Fig. S4). Genetic hitchhiking refers to changes in gene frequency due to selection at associated loci. We found that when host–parasite coevolution maintains diversity at the resistance locus (*Resist*), and the social encounter parameter is intermediate (α high but not maximal), rare neutral tags can become associated with a positively selected helping allele, allowing *Neutral* tag diversity to persist by hitchhiking. Genetic hitchhiking occurs via three steps.

*Step 1:* Extrinsic balancing selection generates tag diversity at *Resist* (*lag* low; *d* not too high). This means that the individuals in the population who are using *Resist* to recognize kin (“*Resist*-choosing individuals”) will *reliably* interact with their genealogical relatives. This generates selection for helping amongst *Resist*-choosing individuals (kin selection). Technically, we elucidated this step in *SI Appendix*, *Appendix 5* by showing that helping frequency increased to approximately fixation (mutation-selection balance) shortly after *Resist* diversity was maximized and without any appreciable increase in linkage disequilibrium.

*Step 2:* In contrast, there will be negligible tag diversity at *Neutral*, because the social encounter parameter (α) is not high enough (a very high social encounter parameter value precludes *Neutral* hitchhiking, because *Neutral* diversity builds up anyway in these cases). This means that the individuals in the population who are using *Neutral* to recognize kin (“*Neutral*-choosing individuals”) will *unreliably* interact with their genealogical relatives unless they happen to have a rare *Neutral* tag. This generates selection for helping amongst *Neutral*-choosing individuals with a rare *Neutral* tag, and selection for defection amongst *Neutral*-choosing individuals with a common Neutral tag (kin selection), which in turn generates linkage disequilibrium between rare *Neutral* tags and helping (*SI Appendix*, *Appendix 5* and Fig. S4). This step reflects the standard tendency for rare *Neutral* alleles to become associated with helping, as described above and in many previous studies (11, 22, 54, 56, 60).

*Step 3:* Overall, the population will therefore initially evolve toward using *Resist* rather than *Neutral* for kin recognition, because *Resist* has more tag diversity (*Result 3*). Furthermore, helping will be favored. However, the selection for the helping allele causes all alleles associated with the helping allele to be indirectly selected (genetic hitchhiking). As explained above, this includes rare *Neutral* alleles, allowing *Neutral* diversity to accumulate (*SI Appendix*, Fig. S4). We note that a small amount of *Trait* and *Choice* mutation (*µ_Trait_*, *µ_Choice_*) is sometimes required for hitchhiking to occur, as explained in *SI Appendix*, *Appendix 6*.

We now briefly give the technical reasons why we were able to pinpoint hitchhiking with the helping allele as the driver of *Neutral* diversity build-up in this area of parameter space (int. α, low *lag*, int./high *d*); see *SI Appendix*, *Appendix 7* for a full account. Readers uninterested in these technical details may skip this paragraph. We proceeded by ruling out all other possible explanations for *Neutral* diversity build-up. First, we found that, when individuals are given the option to use a parasite resistance locus for kin recognition: (i) the linkage disequilibrium between rare neutral tags and helping is decreased; (ii) given this observed decrease in linkage disequilibrium, we proved that the fitness of rare neutral tags does not increase with an increase in the population helping frequency. Taking (i) and (ii) together, this means that the *Neutral* tag diversity build-up is not being driven by an increase in the strength of epistatic coselection of rare *Neutral* tags and the helping allele. Second, we found that rare *Neutral* tags do not enter into linkage disequilibrium with *Resist* alleles, which rules out epistatic interactions between them (which would generate linkage disequilibrium), as well as hitchhiking interactions (which would require linkage disequilibrium), as drivers of the *Neutral* tag diversity build-up. Third, we found that the *Neutral*-choosing *Choice* allele, which enters into linkage disequilibrium with rare *Neutral* tags, does not experience any component of direct positive selection that is not also experienced by rare *Neutral* tags, which means that the *Neutral* tag diversity build-up is not being driven by epistatic or hitchhiking interactions with a *Choice* allele. This process of elimination reveals that hitchhiking with the helping allele is driving the build-up of *Neutral* tag diversity (*SI Appendix*, *Appendix 7*).

The outcome of hitchhiking is that *Neutral* diversity may then evolve to be greater than or equal to *Resist* diversity. If host–parasite coevolution causes appreciable oscillations in *Resist* allele frequencies (higher *d*/*lag*), then *Resist* tags may be worse indicators of relatedness than *Neutral* tags, allowing *Neutral* to hitchhike to a higher tag diversity, and hence be favored as the kin recognition locus. If host–parasite oscillations are less extreme, then *Neutral* and *Resist* can evolve toward equal diversity, such that selection is indifferent with regard to which locus is used for kin recognition (Fig. 6).

These hitchhiking results highlight that there is a region of parameter space where host–parasite coevolution facilitates the long-term maintenance of genetic kin recognition but not because the parasite resistance locus is directly used for kin recognition. Instead, linkage disequilibrium between rare *Neutral* tags and a positively selected helping allele means that host–parasite coevolution can generate tag diversity at a locus that is not directly involved in parasite resistance. This allows host–parasite interactions to continue being governed by the parasite resistance locus, without interference from kin recognition. It similarly allows kin recognition to be underpinned by a locus that is not directly involved in parasite resistance. By retaining separate loci for each phenotype, kin recognition and parasite resistance are uncoupled and can evolve relatively independently. This allows individuals to avoid the cost of increased parasite susceptibility, which arises as a consequence of kin recognition-induced changes in tag frequencies (Fig. 3*B*). The evolution of phenotype uncoupling can therefore be understood through an adaptationist lens as a means by which individuals can simultaneously optimize two different phenotypes that would otherwise be subject to an adaptive trade-off (55).

**Result 5:** *Host–parasite coevolution can cause diversity to build up at an otherwise-neutral recognition locus by genetic hitchhiking, avoiding the cost of increased parasite susceptibility.*

## Discussion

We first examined whether host–parasite coevolution alone can facilitate genetic kin recognition at the locus for parasite resistance. We found that host–parasite coevolution only stabilizes genetic kin recognition when parasites adapt rapidly and virulence is intermediate or high (Result 1; Fig. 3*A*). In addition, because genetic kin recognition can influence the frequency of different alleles, using the resistance locus for kin recognition can increase parasite susceptibility (Result 2; Fig. 3*B*). These results suggested that the extent to which host–parasite coevolution could solve Crozier’s paradox can be relatively limited.

We then examined the consequences of allowing the genetic architecture to evolve, so that natural selection could choose whether recognition was based on the parasite resistance (*Resist*) or an otherwise-neutral (*Neutral*) locus. We found that this allowed genetic kin recognition to be stable over a larger area of parameter space (Result 4; Fig. 5*B*). The reason for this is that if the social encounter parameter is intermediate (α high but not maximal), host–parasite coevolution can maintain tag diversity at another locus, by genetic hitchhiking (Result 5; Fig. 6). Consequently, our results show a way in which host–parasite coevolution can stabilize genetic kin recognition, without susceptibility to parasites being increased.

### Hitchhiking and the Evolution of the Genetic Architecture.

Our results reveal a role for genetic hitchhiking in the evolution of genetic kin recognition. Genetic hitchhiking refers to changes in gene frequency due to selection at associated loci (57, 59). We found that if the social encounter parameter is intermediate (α high but not maximal), then rare alleles at neutral recognition loci can be maintained by hitchhiking on helping alleles that are positively selected as a consequence of extrinsic balancing selection. Direct selection of rare tags at parasite resistance loci may lead to the positive selection of helping, which in turn may lead to the indirect selection of all alleles associated with helping, including rare tags at other recognition loci.

Our results suggest that individuals may often evolve to be indifferent about the particular locus used for recognizing and helping kin, with tag diversity at different loci maintained by genetic hitchhiking. Although we did not examine this explicitly, one possible implication of this indifference is that individuals may evolve to use a mixture of recognition loci. This type of kin recognition, where multiple recognition loci contribute to the identification of kin, rather than just one recognition locus, has been observed in house mice and is postulated in other vertebrates (21, 61). An advantage of multilocus matching is that it may be better at identifying pedigree relatedness, which is what matters for the evolution of multigene adaptations (49, 62–64). Formal theoretical modeling is required to examine the evolution of multi- versus single-locus matching.

Our hitchhiking results emphasize the critical importance of allowing the genetic architecture to evolve (4, 65, 66). Previous theory assumed that a fixed locus had to act as the recognition locus (9, 11). In contrast, we allowed natural selection to “choose” the recognition locus. We found that letting the recognition locus evolve increases the area of parameter space where genetic kin recognition is stable (Fig. 5*B*). By letting the recognition locus evolve, we also found that kin recognition can get started by using a parasite resistance locus for recognition, and then that parasite resistance locus can allow diversity at a neutral locus to build up by hitchhiking, which can then develop diversity and take over as the recognition locus. An advantage of this evolution is that parasite resistance can kickstart the evolution of kin recognition but uncouple itself from kin recognition in the long run, so that parasite resistance and kin recognition do not ultimately constrain each other.

### Kin Recognition and the Red Queen.

Our results suggest that the evolution of genetic kin recognition and sex require particular forms of host–parasite coevolution (Red Queen dynamics). We have shown that genetic kin recognition is most likely to be favored by rapidly adapting parasites with intermediate or high virulence. Sex and recombination are also most likely to be favored when parasites evolve rapidly and have intermediate or high virulence (25–31). In both cases, what matters is that selection does not fluctuate too slowly or too fast, so that resistance alleles are under negative frequency dependence. We directly modeled the outcome of host–parasite coevolution via the rate at which parasites adapt to common genotypes and parasite virulence. An alternative would be to explicitly model the dynamics of a parasite population and vary the basic biological parameters, such as generation times, as well as the form of host–parasite interaction (e.g., gene-for-gene or matching allele). Furthermore, we assumed a constant host population size, but a biologically reasonable extension would be to permit parasite-induced fluctuations. Again, a comparison with models for sex and recombination can be made, where an analogous diversity of approaches has provided complementary results (31, 67).

### Resolutions of Crozier’s Paradox.

Our results suggest that Crozier’s “host–parasite coevolution” resolution to Crozier’s paradox is likely to be of limited importance, because it requires a particular form of host–parasite interaction that cannot be expected to arise generally (rapidly adapting parasites with intermediate or high virulence). Furthermore, in the cases where host–parasite interactions do stabilize tag diversity, we showed that this may be associated with increased parasite susceptibility and consequently reduced fitness. To avoid this fitness cost, we showed that individuals may evolve (via hitchhiking) to use a neutral recognition locus in the long term. A second possible way to avoid this fitness cost, which we did not explicitly consider, could be to switch to using environmental rather than genetic cues to recognize kin (e.g., “grew up in same nest”) (3, 6). These results may explain the lack of empirical support for MHC-based kin recognition (21). Multiple social encounters may be a more promising resolution to Crozier’s paradox, both because it is expected to stabilize kin recognition in a broad parameter space and because it does not generate an additional fitness cost that may lead to selection for switching to a different recognition locus or to environmental cues (22).

We have shown that, in different areas of parameter space, there may be different resolutions of Crozier’s paradox. When parasites are rapidly adapting with intermediate or high virulence, host–parasite coevolution acting alone (i.e., without multiple social encounters) can stabilize genetic kin recognition. Similarly, when the social encounter parameter (α) is high, multiple social encounters acting alone (i.e., without extrinsic balancing selection) can stabilize genetic kin recognition. When the social encounter parameter is intermediate (α high but not maximal), host–parasite coevolution and multiple social encounters can synergistically combine to stabilize genetic kin recognition, first at a parasite resistance locus and then (via hitchhiking) at a neutral locus. Furthermore, there may be other resolutions of Crozier’s paradox, and further synergistic interactions between them, that apply in further unexplored regions of parameter space. The point is not that one particular resolution is “fundamental”—all is required is that there is a mechanism to remove positive frequency dependence (common tag advantage) at the recognition locus, and there may be many different ways to achieve this. Another possibility that deserves further study is whether physical linkage between alleles at the *Tag* and *Trait* loci—known as *greenbeard selection*—can reduce or eliminate common tag advantage (5, 11, 22, 52, 60).

## Conclusion

Our results have shown how Crozier’s paradox can be overturned. We have shown that host–parasite coevolution and multiple interactions can interact synergistically to stabilize genetic kin recognition. This synergism arises through genetic hitchhiking facilitating greater tag diversity. In addition, our results illustrate how the outcome of natural selection can depend critically upon whether the genetic architecture can evolve, emphasizing the importance of allowing for this in theoretical models.

## Materials and Methods

In *SI Appendix*, comprising Appendices 1–8, we provide the full mathematical description of our lifecycle assumptions; mathematical descriptions of various model outputs such as tag diversity, parasite susceptibility & linkage disequilibrium; a derivation of the condition for kin discrimination to be favored; analyses of when genetic kin recognition evolves to be stable; and numerical and analytical evidence for genetic hitchhiking.

## Supplementary Material

Appendix 01 (PDF)

## Data Availability

The data generated during the current study, as well as the Matlab code used to perform numerical calculations, is available at https://doi.org/10.5281/zenodo.8032794 (68). We include programs for implementing our mathematical model and generating figures.
