# Supplementary material for: Host–parasite coevolution and the stability of genetic kin recognition
Source: Proc Natl Acad Sci U S A. 2023 Jul 18;120(30):e2220761120. doi: 10.1073/pnas.2220761120 (PMC10372634; doi:10.1073/pnas.2220761120)
Supplement: Supplementary file 1 — Appendix 01 (PDF) [file pnas.2220761120.sapp.pdf]

Supplementary information for ‘Host-parasite coevolution and the stability of genetic kin recognition’.

This supplementary document comprises the following 8 appendices:

1) **Mathematical model.**

   We construct the mathematical model described in the main text, and mathematically define the various outputs of the model, such as tag diversity, parasite susceptibility, etc.

2) **Derivation of the condition for kin discrimination to be favoured.**

   We identify the area of parameter space where kin discrimination is favoured by kin selection. This is the area of parameter space where, if all available tags are at equal frequency in the population, conditional helping is favoured over defection. Outside of this region of parameter space, conditional helping cannot evolve, precluding kin discrimination based on genetic cues. We therefore focus on this area of parameter space in later appendices when analysing our model.

3) **Analysis of the simplified models where the recognition locus used for kin recognition is fixed (*Scenarios 1* & *2*).**

   We analyse the “fixed recognition locus” scenarios (*Scenarios 1 & 2* in Supplementary Table 1) to identify the area of parameter space where host-parasite coevolution can facilitate the short-term maintenance of genetic kin recognition.

4) **Analysis of the full model where natural selection can choose the recognition locus (*Scenario 3*).**

We analyse the “evolving recognition locus” scenario (*Scenario 3* in Supplementary Table 1) to identify the area of parameter space where host-parasite coevolution can facilitate the long-term maintenance of genetic kin recognition.

5) **A mathematical description of linkage disequilibrium, and how it stabilises genetic kin recognition.**

We give a more in-depth, mathematical explanation of the various ways in which linkage disequilibria facilitate the maintenance of genetic kin recognition.

6) **Effect of mutation.**

We give a more in-depth account of how mutation at the *Trait* and *Choice* loci influence the maintenance of genetic kin recognition.

7) **Evidence for genetic hitchhiking.**

We give a more in-depth account of how we identified ‘hitchhiking with a positively selected helping allele’ as the driver of *Neutral* tag diversity build-up in certain areas of parameter space, and how we ruled out alternative explanations like epistatic selection.

8) **Additional discussion.**

We provide additional discussion of: (*i*) Why should we expect pleiotropy between parasite resistance and genetic kin recognition? (*ii*) The relationship between R_{tag} (relatedness measured at the trait locus) and pedigree relatedness. (*iii*) Why there are many squared terms in our mathematical formulae.

Appendix 1: Mathematical model.

Full model (4-locus model).

We convert our verbal depiction of the lifecycle into genotype frequency recursions, which depict how genotype frequencies change from one generation to the next. We construct the recursions in three parts, with the first part showing how genotype frequencies change as a result of selection, the second part showing how genotype frequencies change as a result of recombination, and the third part showing how genotype frequencies change as a result of mutation.

For an individual who has a given allele i at the *Neutral* locus ($i \in \{1,...,L_{max}\}$), a given allele j at the trait locus (j=0 indicates defection, and j=1 indicates conditional helping), a given allele m at the *Resist* locus ($m \in \{1,...,L_{max}\}$), and a given allele y at the choice locus (y=0 indicates that *Neutral* is chosen as the recognition locus; y=1 indicates that *Resist* is chosen as the recognition locus), we write fecundity as w_{ijmy}. We write the population frequency of individuals with a given genotype *ijmy* as x_{ijmy}. We also make use of the following shorthand notation: $\sum_{j=0}^{1}\sum_{m=1}^{L_{max}}\sum_{y=0}^{1} x_{ijmy} = x_i$ (this is the population frequency of all individuals who have allele i at the *Neutral* locus); $\sum_{i=1}^{L_{max}}\sum_{j=0}^{1}\sum_{y=0}^{1} x_{ijmy} = \tilde{x}_m$ (this is the population frequency of all individuals who have allele m at the *Resist* locus). We also use the notation $\tilde{x}_m^{lag}$ to denote the population frequency of the m allele (at the *Resist* locus) at a previous point in time, *lag* generations ago (note that, in the special case where *lag*=0, $\tilde{x}_m^{lag} = \tilde{x}_m$). Selection causes the frequency of a given genotype *ijmy* to change from x_{ijmy} to x_{ijmy}', according to $x_{ijmy}' = w_{ijmy}x_{ijmy}$, where w_{ijmy} (fecundity) is defined below (Equations A1-A4). We assume that $c \leq 1$ (fecundity loss from helping) and $d \leq 1$ (fecundity loss from being infected by a parasite), which ensures that fecundity is never negative.

The fecundity of an individual who has: a given allele i at the *Neutral* locus; the conditional altruism allele (j=1); a given allele m at the *Resist* locus; the *Neutral*-choosing allele at the choice locus (y=0) allele at the choice locus; can be written as follows (w_{i1m0}):

$$w_{i1m0} = \left(1 + \frac{b\big(\theta + (1-\theta)\sum_{n=1}^{L_{max}} x_{i1n0}\big) - c(\theta + (1-\theta)x_i)}{1 - \alpha(1-x_i)(1-\theta)} + \frac{b(1-\theta)\sum_{l=1}^{L_{max}} x_{l1m1}}{1-\alpha(1-\tilde{x}_m)(1-\theta)} - A\right)\left(1 - d\tilde{x}_m^{lag} + d\sum_{n=1}^{L_{max}} (\tilde{x}_n^{lag})^2\right). \qquad (A1)$$

To make sense of this equation, note that, each generation, an individual with this genotype (*i1m0*) will provide help with probability $\frac{\theta+(1-\theta)x_i}{1-\alpha(1-x_i)(1-\theta)}$, leading to a generational cost of helping of $\frac{c(\theta+(1-\theta)x_i)}{1-\alpha(1-x_i)(1-\theta)}$. Each generation, an individual with this genotype (*i1m0*) may receive help from individuals using *Neutral* as a basis for tag matching, resulting in a generational benefit of $\frac{b\left(\theta+(1-\theta)\sum_{n=1}^{L_{max}} x_{i1n0}\right)}{1-\alpha(1-x_i)(1-\theta)}$. Furthermore, an individual with this genotype (*i1m0*) may receive help from individuals using *Resist* as a basis for tag matching, resulting in a generational benefit of $\frac{b(1-\theta)\sum_{l=1}^{L_{max}} x_{l1m1}}{1-\alpha(1-\tilde{x}_m)(1-\theta)}$. Individuals will also incur a generational cost of competition, given by *A*. *A* does not depend on the genotype of the focal individual (it is the same for all individuals), and its value is such that the average fecundity in the population is equal to 1, as it should, for a population that is neither growing nor shrinking. The explicit equation for *A* is difficult to write down without a lot of complex subscripting, and it isn't particularly illuminating except to convey that it ensures that mean fitness is equal to 1, so we give the following definition for *A* rather than writing it out explicitly: $A \text{ is such that } \sum_{i,j,m,y}\big(w_{ijmy_A} x_{ijmy}\big) = 1$. Summing these benefits and subtracting the costs

gives rise to the first bracketed term in Equation A1. This component of fecundity captures the consequences of social interactions.

The second bracketed term $\left(1 - d\tilde{x}_m^{lag} + d\sum_{n=1}^{L_{max}}(\tilde{x}_n^{lag})^2\right)$ is applied multiplicatively, and captures the consequences of extrinsic selection. It comprises the term $-d\tilde{x}_m^{lag}$, which is the probability of being infected by a parasite ($\tilde{x}_m^{lag}$) multiplied by the fecundity consequence of being infected by a parasite (–*d*). Overall, $-d\tilde{x}_m^{lag}$ gives the fecundity cost of extrinsic selection. The second bracketed term $\left(1 - d\tilde{x}_m^{lag} + d\sum_{n=1}^{L_{max}}(\tilde{x}_n^{lag})^2\right)$ also comprises the term $+d\sum_{n=1}^{L_{max}}(\tilde{x}_n^{lag})^2$, which is applied indiscriminately to all individuals, and captures competitive effects arising from extrinsic selection, and serves to ensure that the population mean fitness remains at 1. This fecundity component serves to increase the relative fitness of individuals with rarer alleles at the *Resist* locus, when allele frequency is measured at some previous point in time, *lag* generations ago.

The fecundity of an individual who has: a given allele *i* at the *Neutral* locus; the conditional altruism allele (*j*=1); a given allele *m* at the *Resist* locus; the *Resist*-choosing allele at the choice locus (*y*=1) allele at the choice locus; can be written as follows (w_{i1m1}). This fecundity function (Equation A2) is similar to Equation A1, other than it is now the frequency of an allele residing at the *Resist* locus (*m*) that dictates whether help is given (i.e. how *c* is weighted) and how much help is received by kin (i.e. how θb is weighted), rather than the frequency of an allele residing at the *Neutral* locus (*i*).

$$w_{i1m1} = \left(1 + \frac{b\left(\theta + (1-\theta)\sum_{l=1}^{L_{max}} x_{l1m1}\right) - c(\theta + (1-\theta)\tilde{x}_m)}{1 - \alpha(1-\tilde{x}_m)(1-\theta)} + \frac{b(1-\theta)\sum_{n=1}^{L_{max}} x_{i1n0}}{1 - \alpha(1-x_i)(1-\theta)} - A\right)\left(1 - d\tilde{x}_m^{lag} + d\sum_{n=1}^{L_{max}}(\tilde{x}_n^{lag})^2\right). \quad (A2)$$

The fecundity of an individual who has: a given allele i at the *Neutral* locus; the defection allele (j=0); a given allele m at the *Resist* locus; either allele at the choice locus (y); can be written as follows (w_{i0m0}). This fecundity function (Equation A3) is similar to Equations A1 & A2, other than, this time, the individual is a defector, so doesn't pay the cost of helping, nor does it receive help from kin.

$$w_{i0my} = \left(1 + \frac{b(1-\theta)\sum_{n=1}^{L_{max}} x_{i1n0}}{1-\alpha(1-x_i)(1-\theta)} + \frac{b(1-\theta)\sum_{l=1}^{L_{max}} x_{l1m1}}{1-\alpha(1-\tilde{x}_m)(1-\theta)} - A\right)\left(1 - d\tilde{x}_m^{lag} + d\sum_{n=1}^{L_{max}} (\tilde{x}_n^{lag})^2\right). \quad (A3)$$

To reiterate, selection takes genotype frequencies from x_{ijmy} to x_{ijmy}', according to $x_{ijmy}' = w_{ijmy}x_{ijmy}$, where the w terms (fecundity functions) are given in Equations A1-A3. Recombination then takes genotype frequencies from x_{ijmy}' to x_{ijmy}'' according to Equation A4 (printed below), where k denotes the alternative allele to j at the trait locus, and z denotes the alternative allele to y at the choice locus. Though Equation A4 looks complicated, it is simply capturing Mendelian inheritance (free recombination) for the case where there are two multiallelic and two biallelic loci to keep track of.

Mutation then takes genotype frequencies from x_{ijmy}'' to x_{ijmy}''' according to Equation A5 (printed below), where k denotes the alternative allele to j at the trait locus, and z denotes the alternative allele to y at the choice locus. We did not allow mutation at candidate recognition loci, because we want to determine when selection can maintain tag diversity.

After mutation, a number of haploid adults are sampled randomly from the haploid juvenile population, such that the adult population remains constant in size over generations.

$$
\begin{aligned}
x_{ijmy}'' = x_{ijmy}' \Bigg(& x_{ijmy}' + x_{ikmy}' + \frac{1}{2}\sum_{l\neq i}^{L_{max}} x_{lkmy}' + \sum_{l\neq i}^{L_{max}} x_{ljmy}' + \sum_{n\neq m}^{L_{max}} x_{ijny}' \\
& + \frac{1}{2}\sum_{n\neq m}^{L_{max}} x_{ikny}' + \frac{1}{4}\sum_{l\neq i}^{L_{max}}\sum_{n\neq m}^{L_{max}} x_{lkny}' + \frac{1}{2}\sum_{l\neq i}^{L_{max}}\sum_{n\neq m}^{L_{max}} x_{ljny}' + x_{ijmz}' + \frac{1}{2}x_{ikmz}' \\
& + \frac{1}{4}\sum_{l\neq i}^{L_{max}} x_{lkmz}' + \frac{1}{2}\sum_{l\neq i}^{L_{max}} x_{ljmz}' + \frac{1}{2}\sum_{n\neq m}^{L_{max}} x_{ijnz}' + \frac{1}{4}\sum_{n\neq m}^{L_{max}} x_{iknz}' \\
& + \frac{1}{8}\sum_{l\neq i}^{L_{max}}\sum_{n\neq m}^{L_{max}} x_{lknz}' + \frac{1}{4}\sum_{l\neq i}^{L_{max}}\sum_{n\neq m}^{L_{max}} x_{ljnz}' \Bigg) \\
& + \sum_{l\neq i}^{L_{max}} x_{ljmy}' \Bigg(\frac{1}{2}x_{ikmy}' + \frac{1}{4}\sum_{n\neq m}^{L_{max}} x_{ikny}' + \frac{1}{4}x_{ikmz}' + \frac{1}{4}\sum_{n\neq m}^{L_{max}} x_{ijnz}' + \frac{1}{2}x_{ijmz}' \\
& + \frac{1}{2}\sum_{n\neq m}^{L_{max}} x_{ijny}' + \frac{1}{8}\sum_{n\neq m}^{L_{max}} x_{iknz}' \Bigg) \\
& + x_{ikmy}' \Bigg(\frac{1}{2}x_{ijmz}' + \frac{1}{2}\sum_{n\neq m}^{L_{max}} x_{ijny}' + \frac{1}{4}\sum_{l\neq i}^{L_{max}}\sum_{n\neq m}^{L_{max}} x_{ljny}' + \frac{1}{4}\sum_{l\neq i}^{L_{max}} x_{ljmz}' \\
& + \frac{1}{4}\sum_{n\neq m}^{L_{max}} x_{ijnz}' + \frac{1}{8}\sum_{l\neq i}^{L_{max}}\sum_{n\neq m}^{L_{max}} x_{ljnz}' \Bigg) \\
& + \sum_{n\neq m}^{L_{max}} x_{ijny}' \Bigg(\frac{1}{2}x_{ijmz}' + \frac{1}{8}\sum_{l\neq i}^{L_{max}} x_{lkmz}' + \frac{1}{4}x_{ikmz}' + \frac{1}{4}\sum_{l\neq i}^{L_{max}} x_{ljmz}' \\
& + \frac{1}{4}\sum_{l\neq i}^{L_{max}} x_{lkmy}' \Bigg) \\
& + x_{ijmz}' \Bigg(\frac{1}{8}\sum_{l\neq i}^{L_{max}}\sum_{n\neq m}^{L_{max}} x_{lkny}' + \frac{1}{4}\sum_{n\neq m}^{L_{max}} x_{ikny}' + \frac{1}{4}\sum_{l\neq i}^{L_{max}}\sum_{n\neq m}^{L_{max}} x_{ljny}'
\end{aligned}
$$

$$+\frac{1}{4}\sum_{l\neq i}^{L_{max}} x_{lkmy}'\Bigg) + \frac{1}{8}\sum_{n\neq m}^{L_{max}} x_{ijnz}'\sum_{l\neq i}^{L_{max}} x_{lkmy}' + \frac{1}{8}x_{ikmz}'\sum_{l\neq i}^{L_{max}}\sum_{n\neq m}^{L_{max}} x_{ljny}'$$

$$+\frac{1}{8}\sum_{n\neq m}^{L_{max}} x_{ikny}'\sum_{l\neq i}^{L_{max}} x_{ljmz}'. \qquad (A4)$$

$$x_{ijmy}''' = x_{ikmy}''\mu_{Trait}(1-\mu_{Choice}) + x_{ijmz}''\mu_{Choice}(1-\mu_{Trait}) + x_{ikmz}''\mu_{Trait}\mu_{Choice}$$
$$+ x_{ijmy}''(1-\mu_{Trait})(1-\mu_{Choice}). \qquad (A5)$$

By combining our selection ($x_{ijmy}\mapsto x_{ijmy}'$), recombination ($x_{ijmy}'\mapsto x_{ijmy}''$) and mutation ($x_{ijmy}''\mapsto x_{ijmy}'''$) equations, we obtain dynamically sufficient recursions, detailing how genotypes change in frequency across a generation ($x_{ijmy}\mapsto x_{ijmy}'''$). We iterate the recursions over successive generations, to elucidate the (quasi) equilibrium genotype frequencies.

We note that, until *lag* generations have passed, $\tilde{x}_m^{lag}$ will be undefined, because until *lag* generations have passed, $\tilde{x}_m^{lag}$ will be referring to the population frequency of a parasite resistance allele in a *negative* generation, which is nonsensical. We assume, in these first *lag* generations, that $\tilde{x}_m^{lag}$ equals zero, which means that we do not apply selection stemming from host-parasite interactions until *lag* generations have passed. This corresponds to the time required for parasites to initially adapt to the nascent host population.

Initial conditions: For each numerical simulation of our population genetic recursions ('run'), we assume that, at a given recognition locus, one tag is initially dominant, and the remaining tags are rare. Specifically, we set the initial frequency of one tag to 0.9, and we randomly distribute the remaining 0.1 amongst the other L_{max}-1 tags (with each tag frequency above zero). We also assume that the helping allele is initially rare (we set the initial helper proportion of

each tag to 0.1). The start point of our runs is therefore largely indiscriminate defection. This allows us to examine the evolution as well as the maintenance of kin discrimination based on genetic cues. It can also be thought of as an unfavourable scenario for the evolution of kin discrimination based on genetic cues (low initial tag diversity). We assume that each *Choice* allele is equally common (set to 0.5 frequency), except when we want to fix the recognition locus at either *Resist* or *Neutral* (no *Choice* evolution), in which case all individuals trivially start with the same *Choice* allele (one allele set to 1 frequency and the other set to 0).

To reiterate, our initial conditions are: each *Choice* allele is set to 0.5; the helping allele is set to 0.1; one tag at each candidate recognition locus is set to 0.9, and the remaining tags are set to some number <0.1 such that the total tag frequency at each locus equals 1. One thing to note here is that many different distributions of genotype frequencies may each satisfy our initialisation criteria. For instance, we could initiate the population with positive linkage disequilibrium between helping and *Resist*-choosing, such that more *Resist*-choosing individuals initially have the helping allele than the defecting allele. Conversely, we could initiate the population with no linkage disequilibrium between helping and *Resist*-choosing, such that an equal proportion of *Resist*-choosing individuals have the helping and defecting alleles. The important thing to note is that our initial conditions do not specify the initial linkage disequilibria. Instead of specifying some arbitrary set of initial linkage disequilibria between the different loci, our general approach is to take multiple iterations over each parameter combination, each with a slightly different distribution of initial genotype frequencies (where each distribution satisfies our initialisation criteria, but is otherwise randomly generated). Results are then given as averages over the (very slight) differences in initial genotype frequencies. Specifically, all heatmaps (Figure 3 / 5 / 6; Supplementary Figure 2 / 3) show average results taken over three numerical iterations of each parameter combination.

Summary statistics: For each run, we track five summary statistics, which we use to describe the equilibrium state of the population. Summary statistics are measured across a given time period, starting at generation $T_{end} - T_{interval}$ and ending at generation T_{end}, where $T_{interval}$ gives the length of the time period, and T_{end} gives the end-point ($T_{interval} \geq 0$ & $T_{end} \geq 1$). The five summary statistics are:

- *Neutral* tag diversity.
- *Resist* tag diversity.
- Frequency of the conditional helping allele.
- Frequency of the *Resist*-choosing allele.
- Parasite susceptibility.

Below, we give their precise mathematical definitions (Equations A6–A10). We take x_{it} to be the population frequency of *Neutral* tag *i* in generation *t.*

First, we calculate the average-over-time-and-over-tags *Neutral* tag frequency. Or more precisely, if an individual (tag not specified) is randomly chosen from a population, from a generation that is randomly chosen from within $T_{end} - T_{interval}$ to T_{end}, we calculate the expected frequency of the individual's tag as $\frac{\sum_{i=1}^{L_{max}} \sum_{t=T_{end}-T_{interval}}^{T_{end}} x_i^2}{1+T_{interval}}$. The average-over-time number of *Neutral* tags is then simply given by the inverse of this. We note that this metric is not the countable number of tags – such a measure would be misleading, because it would give equal weight to tags that are limitingly rare and exceedingly common. Rather, this metric is an effective tag number based on tag frequencies. It is convenient to transform the average-over-time number of *Neutral* tags so that it varies between 0 and 1, with 0 corresponding to one tag at fixation, and 1 corresponding to all of the L_{max} available tags being maintained at equal frequency. This gives us our first summary statistic:

$$Neutral\ Tag\ Diversity\ |_{T_{end},T_{interval}} = \frac{\frac{1 + T_{interval}}{\sum_{i=1}^{L_{max}} \sum_{t=T_{end}-T_{interval}}^{T_{end}} x_{it}^2} - 1}{L_{max} - 1}. \qquad (A6)$$

Resist tag diversity can be obtained, *mutandis mutatis*:

$$Resist\ Tag\ Diversity\ |_{T_{end},T_{interval}} = \frac{\frac{1 + T_{interval}}{\sum_{m=1}^{L_{max}} \sum_{t=T_{end}-T_{interval}}^{T_{end}} \tilde{x}_{mt}^2} - 1}{L_{max} - 1}. \qquad (A7)$$

We note that these diversity measures (Equations A6 & A7) are versions of Simpson's Diversity Index (1). Technically, our Equations A6 & A7 are obtained by taking an average-over-time Simpson's Diversity Index, then taking its inverse, then scaling it so that it varies between a minimum possible value of 0 and maximum possible value of 1 (rather than between 1 and L_{max}). The average frequency of the helping allele can be written as follows:

$$coop|_{T_{end},T_{interval}} = \frac{\sum_{t=T_{end}-T_{interval}}^{T_{end}} \sum_{i=1}^{L_{max}} \sum_{m=1}^{L_{max}} \sum_{y=0}^{1} x_{i1my}}{1 + T_{interval}}. \qquad (A8)$$

The average frequency of the *Resist*-choosing allele can be written as follows:

$$choice|_{T_{end},T_{interval}} = \frac{\sum_{t=T_{end}-T_{interval}}^{T_{end}} \sum_{i=1}^{L_{max}} \sum_{m=1}^{L_{max}} \sum_{j=0}^{1} x_{ijm1}}{1 + T_{interval}}. \qquad (A9)$$

The probability of being infected by a parasite, for a given individual in a given generation, is given by the frequency of its *Resist* allele recorded *lag* generations ago, $\tilde{x}_m^{lag}$. The average

probability of being infected by a parasite (parasite susceptibility) is obtained by taking an average of this over individuals and generations:

$$susceptible|_{T_{end},T_{interval}} = \frac{\sum_{t=T_{end}-T_{interval}}^{T_{end}} \sum_{m=1}^{L_{max}} \tilde{x}_m \tilde{x}_m^{lag}}{1 + T_{interval}}. \qquad (A10)$$

We define equilibrium as the point at which the five summary statistics are no longer changing. At equilibrium, then, $T_{interval}$ and T_{end} are both sufficiently large that, for a further increase in either $T_{interval}$ or T_{end}, there is negligible change in any of the summary statistics. Therefore, at equilibrium, $T_{interval}$ and T_{end} are large, but they are arbitrary insofar that their precise values do not non-negligibly change any of the summary statistics. We can therefore drop the $T_{end}, T_{interval}$ indexing when writing our *equilibrium* summary statistics: $Neutral\ Tag\ Diversity^*$, $Resist\ Tag\ Diversity^*$, $coop^*$, $choice^*$ & $suscept^*$. We obtain these equilibrium summary statistics by iterating our recursions for a sufficiently long period of time, and using a sufficiently large interval to calculate them with respect to (sufficiently large $T_{interval}$ and T_{end}).

All heatmaps (Figure 3 / 5 / 6; Supplementary Figure 2 / 3) were generated using the same *lag* and *d* combinations (*lag* array = 0,10,20,…,100; *d* array = 0,0.1,0.2,…,1), meaning each figure has the same "resolution" (pixel size).

Simplified models

In addition to our full model, where natural selection can choose the recognition locus, we also considered two simplified versions of the model. In the first simplified version of the model (which we call *Scenario 1*), the recognition locus is fixed at an otherwise-neutral locus (*Neutral*). In the second simplified version of the model (which we call *Scenario 2*), the

recognition locus is fixed at a parasite resistance locus (*Resist*). We call the full model *Scenario 3*.

Scenario	Assumptions		Model
	Initial population state	Choice mutation, μ_{Choice}	
(1) Genetic kin recognition without extrinsic selection.	All individuals have the *Choice* allele that makes them use *Neutral* for kin recognition	0	This leads to a 3-locus (*Trait–Neutral–Resist*) model where selection on kin recognition is unaffected by host-parasite interactions, and selection on parasite resistance is unaffected by social interactions.
(2) Genetic kin recognition with extrinsic selection.	All individuals have the *Choice* allele that makes them use *Resist* for kin recognition	0	This leads to a 2-locus (*Trait–Resist*) model where a parasite resistance locus is used for kin recognition.
(3) Genetic kin recognition with an evolving recognition locus.	Both *Choice* alleles are initially present at equal frequency.	≥0	This leads to a 4-locus (*Trait–Choice–Neutral–Resist*) model where either a parasite resistance locus, or an otherwise-neutral locus, is used for kin recognition.

Supplementary Table 1. The three scenarios examined.

<u>*Scenario 1*</u>

In *Scenario 1*, we considered a simplified version of the model where genetic kin recognition is forced to be based on a recognition locus that is otherwise-neutral (no extrinsic selection). To obtain this simplified model, first we take the full model. Then, we assume that all individuals initially have the choice allele that causes them to use *Neutral* as the recognition locus. Furthermore, we set choice mutation to zero (μ_{Choice}=0) so that all individuals continue to use *Neutral* throughout the entire evolutionary process. We still track evolution (genetic diversity) at the parasite resistance locus (*Resist*), as this gives us a baseline level of parasite susceptibility that arises when the resistance locus is not also being used for kin recognition.

These settings simplify the full mathematical model, so that now, individuals have no choice but to use an otherwise-neutral locus for kin recognition, and selection on kin

recognition stems only from social interactions, not host-parasite interactions. Conversely, selection on parasite resistance stems only from host-parasite interactions, not social interactions. Technically, it is a three-locus (*Trait–Resist–Neutral*) model.

A technical point regarding this scenario is that, in finite populations, selection on parasite resistance will introduce spurious changes in the genetics underpinning social interactions (genetic drift), simply because individuals with high parasite resistance will propagate whatever sociality genes they happen to have alongside their parasite resistance genes, leading to fluctuations in the frequency of sociality genes. The converse is also true – in finite populations, selection on social interactions will introduce fluctuations in parasite resistance gene frequency (genetic drift). The increased genetic drift means that, in finite populations, the efficacy of selection on social interactions and host-parasite interactions will be lower when the two systems are operating together (in a three locus *Trait–Resist–Neutral* model) relative to when the systems are operating independently (in a single-locus *Resist* model and a two-locus *Trait–Neutral* model). However, our present model assumes an infinite population, and there is no genetic drift in an infinite population. This means that selection on host-parasite interactions and social interactions will evolve independently in the three locus *Trait–Resist–Neutral* model, equivalently to how they would evolve in respective single-locus *Resist* and two-locus *Trait–Neutral* models.

Scenario 2

In *Scenario 2*, we considered a simplified version of the model where genetic kin recognition is forced to be based on a recognition locus that is also a parasite resistance locus (extrinsic selection). To obtain this simplified model, first we take the full model. Then, we assume that all individuals initially have the choice allele that causes them to use *Resist* as the recognition locus. Furthermore, we set choice mutation to zero (μ_{Choice}=0) so that all individuals continue

to use *Resist* throughout the entire evolutionary process. We do not bother to track evolution at the *Neutral* locus, because it there is trivially no selection acting on it (it is not being used for kin recognition, or anything else).

These settings serve to simplify the mathematical model, so that now, individuals have no choice but to use a parasite resistance locus for kin recognition. Technically, it is a two-locus (*Resist–Trait*) model.

We note that, to calculate the extent to which using *Resist* for kin recognition leads to an increased susceptibility to parasites, relative to when not using *Resist* for kin recognition (plotted in Figure 3b), we simply compared the equilibrium parasite susceptibilities that arise in *Scenarios 1* & *2*.

Additional technical points about population viscosity

Before moving on, we make a few final technical points about our assumptions regarding population viscosity (structure). As described in the main text, we assume an infinite haploid population where, for each social encounter, there is a probability θ of encountering a full clone (identical by descent at all loci), and a probability 1-θ of encountering a non-relative (identical by descent at no loci). One way of achieving this particular pattern of population viscosity would be if, before each social encounter, the population mixed itself up ("resetting" population viscosity to zero), then every individual in the population underwent one round of mitosis. Every individual encountering someone would then have a certain probability (θ) of encountering its mitotic clone, and a remaining probability (1-θ) of encountering a complete non-relative from the wider mixed population. This is just one way in which our specified pattern of population viscosity may be achieved (θ clone encounter rate / 1-θ non-relative encounter rate).

However, in general, our specified pattern of population viscosity (θ clone encounter rate / 1-θ non-relative encounter rate) is unlikely to be very realistic. One reason is that we are only allowing individuals to interact with full clones (identical by descent at all four loci) or complete non-relatives (identical by descent at none of the four loci), whereas in general, individuals may be partially related (identical by descent at one, two or three of the four loci). A second reason is that our pattern of population viscosity is assumed to be constant over time, whereas in general, social groups may become more or less related over time due to migration and other drivers of demographic change. Our intention here is to sacrifice some realism in order to obtain a mathematically tractable description of population viscosity in a four-locus population genetic model. A concerned reader may be comforted by our finding that, in the version of our model where kin recognition is based on an otherwise-neutral locus (*Scenario 1* in Supplementary Table 1), we obtain the same qualitative results to our previous analysis of this scenario in an infinite island model where relatedness can be partial and change over time (Appendix 3) (2).

Appendix 2: Derivation of the condition for kin discrimination to be favoured.

Before analysing the mathematical model, we derive a condition for when kin discrimination will be favoured by kin selection. This condition shows, in cases where each of the L_{max} available tags at a recognition locus are maintained, when tag-based helping (kin discrimination based on genetic cues) confers a greater inclusive fitness return than indiscriminate defection and indiscriminate helping, in a given social interaction. Satisfaction of this condition is a necessary requirement for tag diversity to build up at endogenously evolving recognition loci (*Neutral*), but it is not a sufficient requirement (there also needs to be sufficient partner search, α, or extrinsically maintained tag diversity at another locus to hitchhike on) (2). Satisfaction of this condition is also a necessary requirement for the conditional helping allele to be positively selected, irrespective of whether tag diversity is being maintained extrinsically (*Resist*) or endogenously (*Neutral*).

In the following calculations, we assumed that a focal individual is using the *Neutral* recognition locus for identifying social partners. We did this for notational convenience. The exact same calculations would still hold if the focal individual was conversely using *Resist* (we would just need to change the mathematical notation). More broadly, the calculations hold irrespective of which locus, in the genome, the focal individual is using for kin recognition.

$\underline{IF_{conditional}}$

First, we calculate the inclusive fitness payoff of tag-based (conditional) altruism, which we denote by $IF_{conditional}$. The inclusive fitness payoff of an action is calculated by: (*i*) identifying all individuals (including the actor) affected by the action; (*ii*) weighting each of these individuals according to their genetic relatedness to the actor (similarity at the trait locus); (*iii*)

summing the (relatedness-weighted) fitness consequences of the action across all of the affected individuals (3).

An act of conditional (tag-based) altruism has consequences for: (*i*) the altruist (actor); (*ii*) its recipient (i.e. the altruist's ultimate social partner after the possibility of searching for social encounters); (*iii*) competitors (those who suffer fecundity losses as a result of altruism exhibited by the actor). We note here that the altruist (actor) and its recipient are tag-matched – if they weren't tag-matched, there would be no social interaction (no altruism exhibited), and therefore no fitness effect! However, the altruist (actor) and its competitors may be tag-mismatched.

The relatedness between the altruist (actor) and another individual ('affected individual') is given by:

$$R = \frac{\lambda - \bar{p}}{1 - \bar{p}}, \qquad (A11)$$

where $\bar{p}$ gives the population frequency of the conditional helping allele, and λ gives the probability that the affected individual also has the conditional altruism allele. Equation A11 is a simplified version of Equation 7 in reference (4); see reference (4) for its derivation.

We see by plugging in $\lambda = 1$ that the actor is related to clones of itself by 1 (complete genetic similarity). We see by plugging in $\lambda = \bar{p}$ that, given that competition occurs at the population level (i.e., not locally, or between kin), meaning competitors are *randomly drawn* from the population with respect to the actor, an individual is related to its competitors by zero (unrelated).

To work out the relatedness between the actor (altruist bearing a given tag *i*) and its recipient, we plug in $\lambda = \frac{\theta + (1-\theta)x_i p_i}{\theta + (1-\theta)x_i}$, which is the (per-interaction) probability that the actor's

recipient is also an altruist (recall that θ denotes population viscosity; x_i denotes the population frequency of the actor's tag i; p_i denotes the proportion of individuals bearing the actor's tag i who are altruists). To interpret this expression for λ, note that the denominator gives the per-encounter probability of encountering a tag-matched individual, and the numerator gives the per-encounter probability of encountering a tag-matched altruist. Plugging this into Equation A11, we obtain the following explicit expression for relatedness between actors and their recipients:

$$R_{tag} = \frac{\frac{\theta + (1-\theta)x_i p_i}{\theta + (1-\theta)x_i} - \bar{p}}{1-\bar{p}}. \qquad (A12)$$

Our explicit relatedness expression clarifies that, when an individual is using an exceedingly rare tag to recognise kin ($x_i \to 0$), tag-matching perfectly identifies kin (who, in this model, are clones), leading to maximal relatedness $(R_{tag} = 1)$. When an individual is using a more common tag ($x_i > 0$), tag-matching does not always imply kinship, meaning the tag brings about a reduced relatedness $(R_{tag} < 1)$. When an individual is using a maximally common tag (i.e. a tag at fixation; $x_i = 1$), meaning that everyone in the population is using the actor's tag ($\bar{p} = p_i$), tag-matching brings about a relatedness of θ, which is the relatedness that would arise by interacting with all individuals that are encountered, irrespective of their tag (the tag provides no extra information about relatedness). The relationship between relatedness (R_{tag}) and tag frequency (x_i) is plotted in Figure 4b.

A notable feature of the relatedness expression (Equation A12) is that, if altruists are evenly distributed across tags (no linkage disequilibrium), such that $p_i = \bar{p}$, cooperator frequency $(p_i, \bar{p})$ 'drops out' of the expression for relatedness. This leads to a relatedness of

$$R_{tag}|_{p_i=\bar{p}} = \frac{\theta}{\theta + (1-\theta)x_i}, \qquad (A13)$$

which is invariant (unchanging) with respect to the frequency of the conditional altruism allele $(p_i, \bar{p})$.

Let us assume that each of the L_{max} available tags are held at equal frequency in the population (we relax this assumption when solving our population genetic model; see the other appendices), and that there is no linkage disequilibrium (i.e. $p_i = \bar{p}$). This means that each tag is at the population frequency $1/L_{max}$. Furthermore, it means that, if there are more available tags (increased L_{max}), each given tag is rarer in the population, resulting in a higher relatedness between altruists and their recipients (R_{tag}). Specifically, if each of the L_{max} tags are maintained at equal frequency, relatedness at equilibrium is given by:

$$R_{tag}|_{p_i=\bar{p},\, x_i={}^1/_{L_{max}}} = \frac{\theta}{\theta\ + (1-\theta)\ {}^1/_{L_{max}}}. \qquad (A14)$$

This would result in an inclusive fitness payoff of conditional (tag-based) altruism, on the assumption that tags have evolved to obtain equal frequencies ($p_i = \bar{p}$) and helper proportions ($x_i = {}^1/_{L_{max}}$), of $IF_{conditional} = R_{tag}|_{p_i=\bar{p},\, x_i={}^1/_{L_{max}}}\, b - c$. Using Equation A14, this can be written explicitly as:

$$IF_{conditional} = \frac{\theta}{\theta\ + (1-\theta)\ {}^1/_{L_{max}}} b - c. \qquad (A15)$$

We reiterate that Equation A15 gives the inclusive fitness payoff of kin discrimination when all tags are held at equal frequency. Of course, tags may not be at equal frequency, which

would lead to a different expected inclusive fitness payoff from tag-based altruism. We are focussing on the case where tags have equalised in frequency because we are searching for a necessary, rather than sufficient, condition for kin discrimination based on genetic cues to be favoured. Specifically, the necessary condition is that, when tags have equalised in frequency, conditional altruism based on tag-matching results in a higher inclusive fitness return than indiscriminate defection or indiscriminate cooperation. The condition is not "sufficient" because, even if it is satisfied, the tags may not equalise in frequency in the first place.

IF_{defection}

Having derived, under the assumption that tags have obtained equal frequencies and helper proportions, the inclusive fitness payoff of conditional altruism (kin discrimination based on genetic cues), we can now ask when conditional altruism will be favoured over defection. The inclusive fitness payoff of defection is zero, by definition, as it is a non-social trait (3, 5). This means that conditional (tag-based) helping will be favoured over defection whenever the following condition is satisfied (obtained by evaluating $IF_{conditional} > IF_{defection}$). Failure to satisfy this condition implies that defection will persist at evolutionary equilibrium. The condition is a form of Hamilton's Rule.

$$\frac{\theta}{\theta + (1-\theta)\, {}^{1}/_{L_{max}}} b > c. \qquad (A16)$$

IF_{indiscriminate}

To derive the inclusive fitness payoff for indiscriminate altruism ($IF_{indiscriminate}$), we assume that there is one single tag at fixation (no tag diversity; $x_i = 1, \bar{p} = p_i$). We evaluate our relatedness

coefficient (Equation A12) under this assumption of no tag diversity, which gives $R_{tag}|_{x_i=1} = \theta$. This leads to an inclusive fitness payoff of indiscriminate altruism of:

$$IF_{indiscriminate} = \theta b - c. \qquad (A17)$$

Conditional (tag-based) helping confers a greater inclusive fitness return than indiscriminate altruism whenever $IF_{conditional} > IF_{indiscriminate}$. Using Equations A15 & A17, we can evaluate this condition, and we see that it holds whenever there is diversity at the recognition locus (L_{max}>1). This is intuitive – by discriminating who it interacts with, an individual is more likely to interact with kin, meaning there is a greater inclusive fitness return from cooperating. Therefore, as long as individuals are capable of differentiating individuals based on their tag (L_{max}>1), kin discrimination based on genetic cues will confer a greater inclusive fitness payoff, per social interaction, than indiscriminate altruism. Of course, this conclusion assumes that there is no cost to discriminating – readers are directed to reference (2) for an analysis of how a cost of discriminating influences the evolution of kin discrimination based on genetic cues.

This analysis reveals that, if tags have equalised in frequency and helper proportion, kin discrimination based on genetic cues confers a greater inclusive fitness return, per social interaction, than: (*i*) indiscriminate defection, whenever the condition in Equation A16 is satisfied; (*ii*) indiscriminate helping, whenever there is tag diversity (L_{max}>1). Equation A16 is referred to in the main text with the simpler notation $R_{tag}\, c - b > 0$.

Satisfaction of Equation A16 is a necessary rather than a sufficient condition for the evolution of kin discrimination based on genetic cues. For genetic kin recognition to evolve, there also needs to be negative frequency dependence at the recognition locus, which may arise

if there is sufficient partner search (α) or extrinsic balancing selection stemming from host-parasite interactions.

Conversely, if there is positive frequency dependence at the recognition locus, meaning tag diversity is lost, an indiscriminate strategy will evolve. If $\theta b<c$ (where θ is the relatedness that would arise by choosing social partners at random), this strategy will be indiscriminate defection; if $\theta b>c$, this strategy will be indiscriminate altruism.

Appendix 3: Analysis of the simplified models where the recognition locus used for kin recognition is fixed (*Scenarios 1 & 2*).

In this appendix, we examine whether host-parasite coevolution facilitates genetic kin recognition when the recognition locus is fixed (cannot evolve). To address this, we determined when genetic kin recognition evolves when individuals can only use an otherwise-neutral recognition locus for genetic kin recognition (no extrinsic selection; *Scenario 1* in Supplementary Table 1). This gave us a baseline amount of genetic kin recognition that evolves in the absence of extrinsic selection. We then determined when genetic kin recognition evolves when individuals can only use a recognition locus that has an additional role in parasite resistance for genetic kin recognition (*Scenario 2* in Supplementary Table 1). By comparing *Scenarios 1 & 2* we could determine, assuming that the recognition locus is fixed, when host-parasite coevolution facilitates genetic kin recognition. The assumption that the recognition locus is fixed will hold relatively well across short evolutionary timescales, meaning these results show when genetic kin recognition is maintained in the evolutionary short term (before the underlying genetic architecture has had time to evolve).

Genetic kin recognition without parasites (*Scenario 1*).

We first examined the scenario where genetic kin recognition can only be based on a locus that is neutral aside from its role in kin recognition (endogenously evolving recognition locus; no extrinsic selection; *Scenario 1* in Supplementary Table 1). This acts as a ‘control’ scenario, where host-parasite coevolution and social evolution do not interfere with each other.

In Appendix 2, we showed that genetic kin recognition is stable if two conditions are met (Supplementary Figure 1). First, kin discrimination must be favoured by kin selection. By

this, we mean that conditional helping (help if matching tag) must have a higher fitness payoff than both defection (never help) and indiscriminate helping (always help, irrespective of tag). For our lifecycle assumptions, this occurs when Hamilton's rule is met, giving $R_{tag}\, b > c$, where R_{tag} is the relatedness between actors and their (tag-matched) social interactants (Supplementary Figure 1) (2–14). R_{tag} is maximal (equal to 1) when individuals are using a limitingly rare tag to identify kin, and decreases to a minimum as the frequency of the tag used to identify kin increases to 1. The minimum value of R_{tag} is given by the baseline population structure (θ), which will be equal to zero in well-mixed populations (Figure 4b).

Second, rare tags must be maintained in the population, so that there is sufficient genetic diversity at the recognition locus (*Neutral*) to allow genetic kin recognition. For our lifecycle assumptions, this occurs when individuals can search for social partners (high α), and there is sufficient mutation at the *Trait* locus (μ_{Trait}) (2). Note that the μ_{Trait} requirement is easy to satisfy in practice, as there only needs to be a very small (or sometimes zero) amount of *Trait* mutation before genetic kin recognition can be stabilised. The explanation for why *Trait* mutation affects the stability of genetic kin recognition is subtle, so we do not give it here. Instead, we return to the question of how mutation affects genetic kin recognition in Appendix 6.

If partner search (α) is high enough, this means that individuals with rare tags can find individuals with the same tag and receive as much help as individuals with common tags. This means that common tags do not increase in frequency, as assumed by Crozier's paradox, and so genetic variability is not eliminated at the recognition locus (Supplementary Figure 1) (2).

Instead, if α is high enough, rather than being eliminated as predicted by Crozier, genetic variability actually *builds up* at the recognition locus (Supplementary Figure 1). This is caused by coevolution between helping and kin recognition (15). As tags become more common, they will become less useful cues of the individual's common ancestry, and so kin

selection is less likely to favour the helping of tag-matched individuals (Figure 4). Consequently, defection can invade at common tags. In contrast, rare tags will be good indicators of relatedness, and so kin selection will favour the helping of tag-matched individuals. This means that rare tags cannot be invaded by defectors. Technically, a statistical association between genes for helping and rare tags builds up (linkage disequilibrium; Supplementary Figure 4a; Appendix 5). The consequence of this coevolution is that individuals with rare tags will have a greater average payoff from social interactions, meaning rare tags increase in frequency, maintaining tag diversity (2, 15, 16).

Overall, in the absence of extrinsic selection, genetic kin recognition evolves when θ (population viscosity) and L_{max} (number of available tags) are sufficiently high that kin discrimination is favoured by kin selection, and α (partner search) and μ_{Trait} (*Trait* mutation) are sufficiently high that rare tags can gain an advantage over common tags, maintaining tag diversity (Supplementary Figure 1). Partner search (α) often needs to be very high before tag diversity is maintained (e.g., α>0.99), but this is likely to be met in species where individuals can move around and choose who to interact with (2). Overall, our results agree with our previous analyses of an analogous scenario, but in an infinite island model (2). Consequently, the conclusion that multiple social encounters (partner search) can eliminate Crozier's paradox appears to be robust to different lifecycle assumptions.

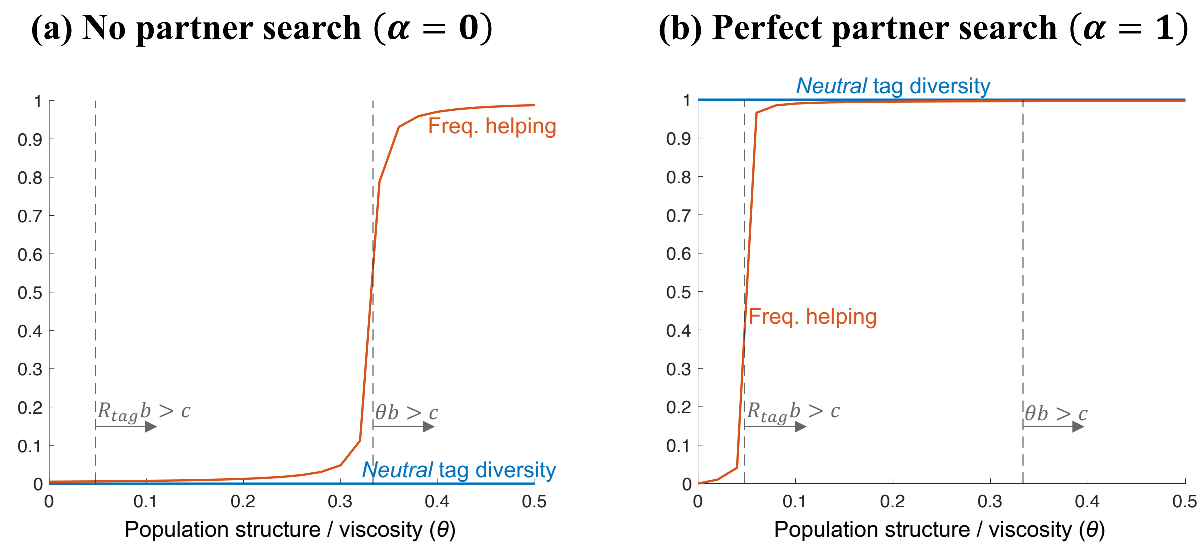

Supplementary Figure 1. When the recognition locus is fixed at an otherwise-neutral locus (Scenario 1), genetic kin recognition only evolves if partner search is high and kin discrimination is favoured by kin selection. We plot the equilibrium: tag diversity (blue lines); population frequency of the conditional helping allele (red lines). Panel a plots results for no partner search (α=0), and panel b plots results for perfect partner search (α=1). The grey dashed vertical lines show when kin discrimination is favoured by kin selection (R_{tag} *b* > c; leftward lines), and when indiscriminate helping is favoured by kin selection (*θ b > c;* rightward lines). Genetic kin recognition (tag diversity + helping) only evolves if partner search is high and kin discrimination is favoured by kin selection (i.e., in panel b to the right of the R_{tag} b > c line). We assumed: *b*=0.3, *c*=0.1, μ_{Trait}=0.0005, L_{max}=10, *θ*=0.25.

<u>Genetic kin recognition with parasites (*Scenario 2*).</u>

We then examined the scenario where genetic kin recognition can only be based on a locus that has an additional role in parasite resistance, and is therefore under extrinsic selection (*Scenario 2* in Supplementary Table 1). We focused on the area of parameter space where R_{tag} *b* > *c* is satisfied, and so kin discrimination is favoured if tag diversity can be maintained (Appendix 2). We started by assuming that partner search (α) is zero, to see when genetic kin recognition can be stabilised by host-parasite coevolution alone.

We found that, when partner search is zero (α=0), genetic kin recognition is only stable if parasites evolve rapidly to better infect common genotypes (low *lag*) and have intermediate virulence (*d*) (Supplementary Figure 2a). Rapid parasite evolution (low *lag*) facilitates tag diversity because, if parasites evolve rapidly to the currently most common tag in the population (low *lag*), this means that rare tags are more likely to have an advantage over common tags in any given generation. Conversely, slow parasite evolution (high *lag*) means that, in any given generation, parasites are less likely to be targeting the most common tags – instead, they will be targeting tags that were common in some previous generation, but which may not be common any more. This means that common tags are more likely to run away to fixation, eliminating tag diversity.

Intermediate virulence (*d*) facilitates tag diversity because, if *d* is too low, the force of extrinsic balancing selection is too weak to overturn Crozier's paradox and stabilise tag diversity. Conversely, if *d* is too high, tags are more likely to cycle severely in frequency, because any tags that are selected in one generation are likely to shoot rapidly up in frequency. The tag frequency oscillations associated with high parasite virulence (*d*) serve to destabilise tag diversity. However, if parasites adapt instantaneously (*lag*=0), tag diversity can be stabilised even for very high parasite virulence (setting *lag*=0 removes the upper limit on *d*) (Supplementary Figure 2a). Rousset & Roze (17) found that increasing parasite virulence (*d*) increases the likelihood that tag diversity is maintained at a parasite resistance locus (no upper limit on *d*), but they were implicitly considering the *lag*=0 case. We therefore recover Rousset & Roze's (17) result as a special case in our broader framework where *lag* can vary.

Increasing the partner search (α) increases the region of parameter space (values of *lag* and *d*) where tag diversity is maintained (Supplementary Figure 2a). The reason for this is that partner search (α) generates an additional advantage for rare tags, as we described in the previous section. However, in some regions of parameter space, characterised by parasites that

adapt slowly (low *lag*) or have high virulence (high *d*), Red Queen dynamics (tag frequency oscillations) can be so severe that, even for maximal partner search (α=1), tag diversity cannot be maintained (Supplementary Figure 2a).

<u>Comparison</u>

We summarised and compared *Scenarios 1 & 2* in Supplementary Figure 2a. A comparison of these scenarios allowed us to determine the extent to which host-parasite coevolution can favour kin recognition at the parasite resistance locus (*Resist*; when the choice of recognition locus cannot evolve).

We found that host-parasite coevolution only facilitates genetic kin recognition in a limited area of parameter space (area *iii* in Supplementary Figure 2a). Specifically, when partner search (α) is low, disfavouring diversity at an otherwise-neutral recognition locus, but parasites are rapidly adapting with intermediate virulence, favouring diversity at a parasite resistance locus.

We also found that host-parasite coevolution can sometimes make genetic kin recognition less likely to be favoured (area *ii* in Supplementary Figure 2a). Specifically, a parasite resistance locus can generate less tag diversity than can be obtained at an otherwise-neutral locus. This occurs when partner search (α) is high, favouring diversity at an otherwise-neutral recognition locus, but parasites are virulent and slowly adapting, leading to wild allele fluctuations and loss of diversity at a parasite resistance locus. This demonstrates that host-parasite coevolution can be a destabilising force on genetic kin recognition, rather than a stabilising force as is commonly assumed (17–21).

<u>Implications for parasite susceptibility.</u>

We examined how parasite susceptibility / resistance is affected by using a parasite resistance locus for genetic kin recognition. To do this, we determined the equilibrium *Resist* diversity and average parasite susceptibility when the parasite resistance locus is being used for kin recognition (*Scenario 2*), and compared this to the equilibrium *Resist* diversity and average parasite susceptibility when the parasite resistance locus is not used for genetic kin recognition (*Scenario 1*).

We find that, when partner search (α) is very high / maximal, social interactions lead to negative frequency dependence at the recognition locus, increasing genetic variability. Therefore, when partner search (α) is very high / maximal, using a parasite resistance locus for kin recognition increases genetic variability at the resistance locus, which reduces parasite susceptibility. This shows that multiple social encounters can interact synergistically with host-parasite interactions to decrease susceptibility to parasites.

Conversely, when partner search (α) is low, social interactions lead to positive frequency dependence at the recognition locus, decreasing genetic variability. Therefore, when partner search (α) is low, using a parasite resistance locus for kin recognition reduces genetic variability at the resistance locus, which increases parasite susceptibility.

If we focus on the area of parameter space where host-parasite coevolution facilitates the short-term maintenance of genetic kin recognition, given by area *iii* in Supplementary Figure 2a, we find that parasite susceptibility increases (either appreciably or negligibly) as a consequence of using the resistance locus for kin recognition. This is because, in this region of parameter space, multiple social encounters and host-parasite coevolution are opposing each other, with the former reducing genetic variability and the latter increasing genetic variability. Therefore, using host-parasite coevolution to stabilise kin recognition comes at the possible cost of reduced performance in host-parasite interactions (adaptive trade-off) (22).

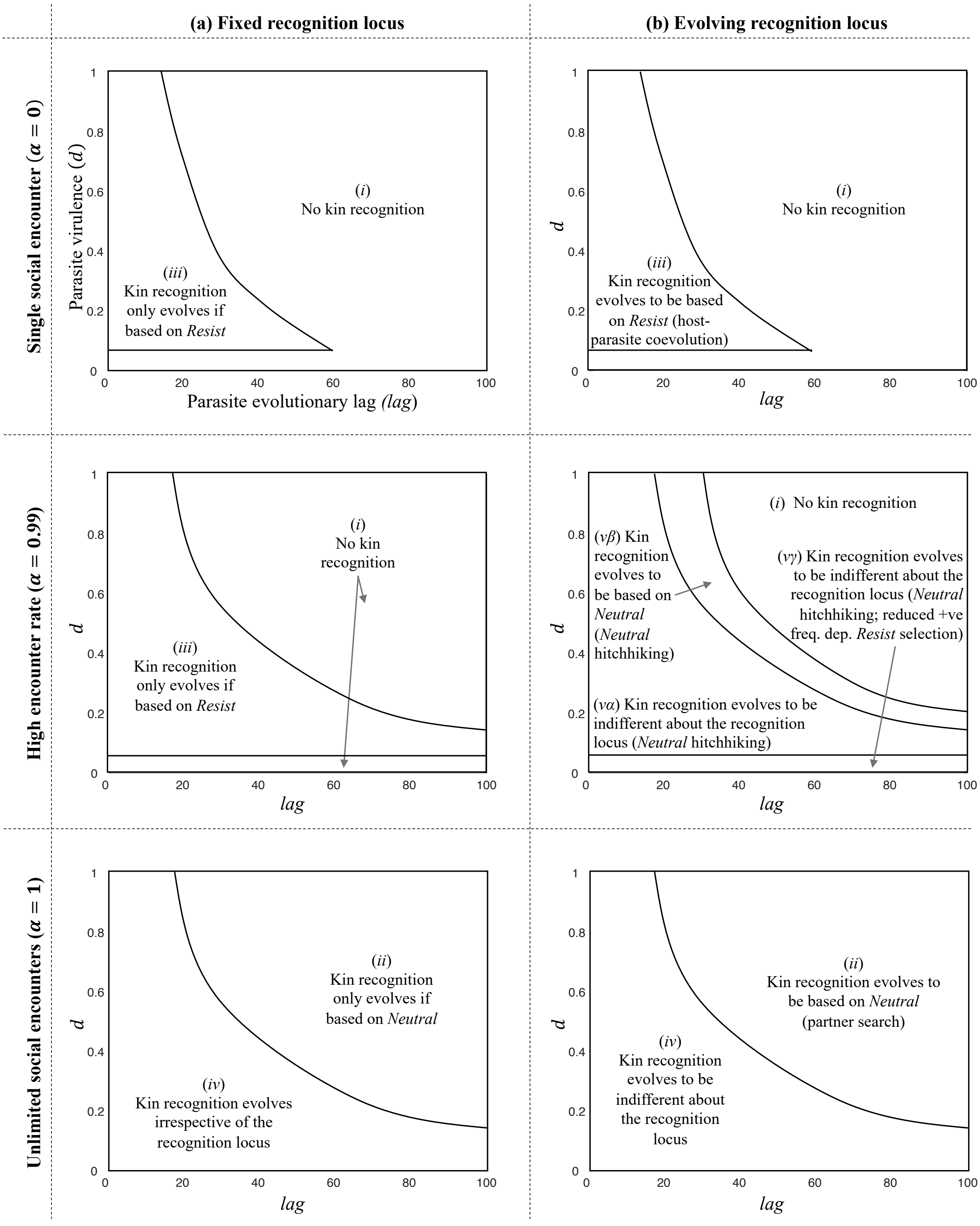

Supplementary Figure 2. Genetic kin recognition when the recognition locus is fixed or evolving. We provide an overview for a region of parameter space where kin discrimination is favoured by kin selection ($R_{tag}\, b - c > 0$), examining when tag diversity is maintained (kin recognition favoured), and at which locus (*Resist* and / or *Neutral*). (**A**) The recognition locus is fixed at either *Neutral* or *Resist* (*Scenarios 1 & 2*). The different areas of parameter space represent cases where

genetic kin recognition is: (*i*) not stable (insufficient diversity at either locus); (*ii*) favoured if based on *Neutral*, but not if based on *Resist* (host-parasite coevolution attenuates kin recognition); (*iii*) favoured if based on *Resist*, but not if based on *Neutral* (host-parasite coevolution facilitates kin recognition); (*iv*) favoured irrespective of whether based on *Neutral* or *Resist*. (**B**) The recognition locus can evolve (*Scenario 3*). Kin recognition is ultimately favoured at the locus where the equilibrium tag diversity is highest. Genetic kin recognition is: (*i*) not stable (insufficient diversity at either locus); (*ii*) favoured at *Neutral*; (*iii*) favoured at *Resist*; (*iv*) favoured at either locus (*Resist* & *Neutral* have equal equilibrium tag diversity); (*v*) supported by genetic hitchhiking. The figure annotations are elaborated in Appendix 3 & 4. We assumed: b=0.3, c=0.1, μ_{Trait}=0.001, L_{max}=10, θ=0.25; (**B**) μ_{Choice}=0.001 (same as Figure 3 / 5 / 6).

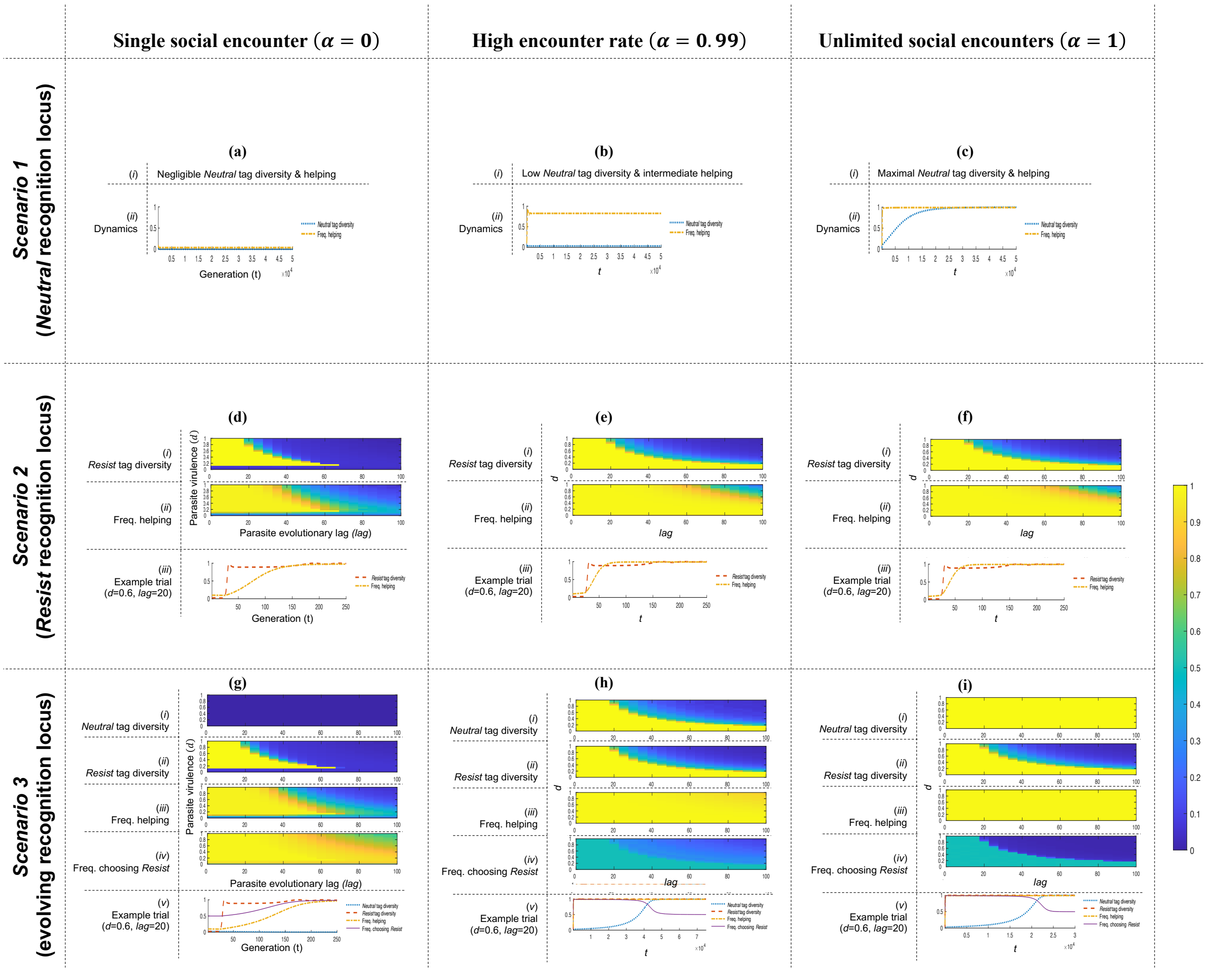

Supplementary Figure 3. Genetic kin recognition when the recognition locus is fixed at a parasite resistance locus (*Scenarios 1 & 2*) or evolving (*Scenario 3*). Example results for a region

of parameter space where kin discrimination is favoured by kin selection ($R_{tag}\, b > c$). (**A–C**) Genetic kin recognition when the recognition locus is fixed at an otherwise-neutral recognition locus (*Neutral*). We record the tag diversity and population frequency of the conditional helping allele, both (*i*) at equilibrium (these results are independent of parasite characteristics, *d* & *lag*), and (*ii*) over time. (**D–F**) Genetic kin recognition when the recognition locus is fixed at a parasite resistance locus (*Resist*). We plot the equilibrium: (*i*) tag diversity; (*ii*) population frequency of the conditional helping allele. Panels *iii* provide examples of single trials, taken from the *lag*=20, *d*=0.6 coordinates in panels *i*-*ii*. (**G–I**) Genetic kin recognition when the recognition locus is evolving. We plot the equilibrium: (*i*) tag diversity at *Neutral* locus; (*ii*) tag diversity at *Resist* locus; (*iii*) population frequency of the conditional helping allele; (*iv*) population frequency of the allele that chooses *Resist* rather than *Neutral* to be the recognition locus. Panels *v* provide examples of single trials, taken from the *lag*=20, *d*=0.6 coordinates in panels *i*-*iv*. The helping allele goes approximately to fixation whenever appreciable tag diversity is maintained. **H** and **B** are equivalent scenarios except that the recognition locus can evolve to be a parasite resistance locus in **H** but not **B**. The maintenance of *Neutral* diversity in **H** but not **B** is due to genetic hitchhiking. We assumed: b=0.3, c=0.1, μ_{Trait}=0.001, L_{max}=10, θ=0.25; (**G–I**) μ_{Choice}=0.001 (same as Figure 3 / 5 / 6).

Appendix 4: Analysis of the full model where natural selection can choose the recognition locus (*Scenario 3*).

In this appendix we examine whether letting the choice of recognition locus evolve increases the area of parameter space where genetic kin recognition is stable. To address this, we determined when genetic kin recognition evolves when individuals have a choice of recognition locus, between an otherwise-neutral locus and a parasite resistance locus. This corresponds to *Scenario 3* in Supplementary Table 1. By comparing *Scenario 3* with *Scenarios 1 & 2* we could determine, without assuming that the recognition locus is fixed, when host-parasite coevolution facilitates genetic kin recognition. This let us examine the consequences of letting the genetic architecture evolve. The assumption that the recognition locus is fixed will not hold very well over long evolutionary timescales, so these results show when genetic kin recognition is maintained in the evolutionary long term.

We found in our analysis of *Scenario 3* that individuals evolve towards using the recognition locus that has the most diversity. In the evolutionary long term, individuals therefore use the candidate recognition locus that maintains the most tag diversity. If equal tag diversity is maintained at each candidate recognition locus, individuals are indifferent with regards to which locus is used (Supplementary Figure 3). This makes intuitive sense – it is better to use the locus with the most tag diversity, because this allows more precise kin recognition (Figure 4).

This result, that individuals evolve to use the recognition locus that maintains the most tag diversity (given as *Result 3* in the main text), is a direct prediction of Hamilton's Part 2 paper introducing inclusive fitness (5). Hamilton noticed that patterns of kin discrimination in insects and birds, regarding when kin discrimination is present and when it is absent, seem to support the following theoretical principle: "The situations which a species discriminates in its

social behaviour tend to evolve and multiply in such a way that the coefficients of relationship involved in each situation become more nearly determinate" (5, 15). In other words, Hamilton's prediction was that, in social situations where the relatedness between altruists and their recipients is variable, there will be selection for more precise kin discrimination, which in turn reduces the variability in relatedness associated with social interactions of that type (relatedness becomes more determinate), such that altruism is increasingly directed towards closer relatives. We found that, through the evolution of the choice locus, genetic kin recognition evolves to become more precise, resulting in less variability in relatedness between actors and their recipients. Therefore, despite the genetic complexities of our model, Hamilton's empirically motivated prediction shines through (15).

There are several outcomes of the *Scenario 3* model. First, we describe what these different outcomes are and what regions of parameter space they arise in (Sections A-E below). These results are summarised Supplementary Figure 2b, and the underlying data is given in Supplementary Figure 3*g*, *h* & *i*. After describing all the different possible outcomes, we will return to the question of whether letting the choice of recognition locus evolve increases the area of parameter space where genetic kin recognition is stable. We will end with an aside regarding the parameter values chosen to generate Figure 1B in the main text. We focused on the area of parameter space where $R_{tag}\, b > c$ is satisfied, and so kin discrimination is favoured if tag diversity can be maintained (Appendix 2).

A. Tag diversity is not maintained, meaning genetic kin recognition is not stable (Supplementary Figure 2b area *i*).

If partner search (α) is too low, it cannot stabilise tag diversity at *Neutral*. If parasites are virulent and adapt slowly (low α, high *lag* & *d*), severe red queen dynamics arising from virulent and poorly adapted parasites generate large allele frequency oscillations at *Resist*,

ultimately destabilising tag diversity at this locus. Alternatively, with low parasite virulence (*d*), the parasite-induced selection pressure on host parasite resistance is too low to stabilise tag diversity at *Resist*. Therefore, if partner search (α) is too low, and parasites are either too weak (low *d*) or too virulent and maladapted (high *lag* & *d*), genetic kin recognition will not evolve.

In these cases, host-parasite coevolution has no effect on whether genetic kin recognition is favoured.

<u>B. Genetic kin recognition is based on *Neutral*, with tag diversity maintained by partner search (α) (Supplementary Figure 2b area *ii*).</u>

In cases where partner search (α) is high, and parasites are too virulent and maladapted (high *lag* & *d*), tag diversity builds up at *Neutral* but not *Resist*. This is because high partner search (α) stabilises tag diversity at *Neutral*, but severe red queen dynamics arising from virulent and poorly adapted parasites generate large allele frequency oscillations at *Resist*, reducing rather than increasing tag diversity. Consequently, individuals evolve towards using *Neutral* for kin recognition.

In this case, host-parasite coevolution has no effect on whether genetic kin recognition is favoured.

<u>C. Genetic kin recognition is based on *Resist*, with tag diversity maintained by host-parasite interactions (α) (Supplementary Figure 2b area *iii*).</u>

In cases where partner search (α) is low, and parasites are adapted with intermediate virulence (low *lag*, intermediate *d*), tag diversity initially builds up at *Resist* but not *Neutral*. This is because partner search (α) is too low to stabilise tag diversity at *Neutral*, but host-parasite coevolution (extrinsic balancing selection) stabilises tag diversity at *Resist*. In these scenarios,

partner search (α) is too low for tag diversity to subsequently accumulate at *Neutral* by genetic hitchhiking (see below). Therefore, individuals evolve to use *Resist* for kin recognition.

In this case, host-parasite coevolution facilitates the long-term maintenance of genetic kin recognition, because it maintains tag diversity at the *Resist* locus.

D. Genetic kin recognition is based initially on *Resist*, but ultimately on either recognition locus (indifferent), with tag diversity at *Neutral* maintained by partner search (Supplementary Figure 2b area *iv*).

In cases where partner search (α) is high, and parasites are not too virulent and poorly adapted (high α, low *lag*, low *d*), maximal tag diversity will be favoured at both *Neutral* and *Resist* (L_{max} tags maintained at both loci at equilibrium). This is because high partner search (α) stabilises tag diversity at *Neutral*, and this in combination with host-parasite interactions (extrinsic balancing selection) stabilises tag diversity at *Resist*. However, in the run-up to equilibrium, tag diversity will build up more quickly at *Resist* than *Neutral* due to the extra stabilising push of extrinsic balancing selection. In these scenarios, the population will switch towards using *Resist* initially, though once maximal tag diversity has been attained at both candidate recognition loci, the population will be indifferent with regards to which candidate recognition locus is used (neutral choice evolution).

In these scenarios, host-parasite coevolution may facilitate the pre-equilibrium, but not the equilibrium, maintenance of genetic kin recognition. The reason is that, in these cases, host-parasite coevolution generates tag diversity more quickly than is attained in the absence of host-parasite coevolution, meaning parasite resistance loci are initially used for kin recognition. But the resulting amount of tag diversity is the same, irrespective of whether there are host-parasite interactions, meaning host-parasite interactions do not affect whether genetic kin recognition is maintained at equilibrium.

E. Genetic kin recognition is initially based on *Resist*, but ultimately based on either *Neutral* alone or on either recognition locus (indifferent), with tag diversity at *Neutral* maintained by hitchhiking (Supplementary Figure 2b area *v*).

Host-parasite coevolution can cause diversity to build up at an otherwise-neutral recognition locus (*Neutral*), by *genetic hitchhiking*, even though that locus (*Neutral*) is not directly involved in parasite resistance. Genetic hitchhiking arises when parasites are adapted and not too virulent (*lag* low, *d* not too high), and partner search (α) is high but not exceedingly high (intermediate). In these cases, tag diversity initially builds up at *Resist* (extrinsic balancing selection) but not at *Neutral* (insufficient partner search). However, this isn't the end of the evolutionary process. Tag diversity subsequently builds up at *Neutral* by *genetic hitchhiking* (23–25). Below, we describe how genetic hitchhiking comes about. Readers interested in how we know genetic hitchhiking is causing the build-up of *Neutral* tag diversity, rather than, say, epistatic selection, should consult Appendix 7.

First, extrinsic balancing selection generates tag diversity at *Resist* (*lag* low, *d* not too high). This means that the individuals in the population who are using *Resist* to recognise kin ('*Resist*-choosing individuals') will *reliably* interact with their genealogical relatives. This generates selection for helping amongst *Resist*-choosing individuals (kin selection). Conversely, insufficient partner search (α) means that there is negligible tag diversity at *Neutral*. This means that the individuals in the population who are using *Neutral* to recognise kin ('*Neutral*-choosing individuals') will *unreliably* interact with their genealogical relatives, unless they happen to have a rare *Neutral* tag. This generates selection for helping amongst *Neutral*-choosing individuals with a rare *Neutral* tag, and selection for defection amongst *Neutral*-choosing individuals with a common Neutral tag (kin selection), which in turn

generates linkage disequilibrium between rare *Neutral* tags and helping (Appendix 5; Supplementary Figure 4c).

Meanwhile, the population evolves towards using *Resist* rather than *Neutral* for kin recognition, because *Resist* has more tag diversity. This increases the proportion of *Resist*-choosing individuals in the population, which accordingly increases the overall strength of selection on the helping allele. The strength of selection on the helping allele increases simply because helping is favoured amongst all *Resist*-choosing individuals, but is only favoured amongst a subset of *Neutral*-choosing individuals (i.e., the ones with rare *Neutral* tags). An increase in the proportion of *Resist*-choosing individuals therefore means that helping is selected across a greater overall fraction of the population, increasing the overall strength of selection on helping.

The (increasingly strong) selection of the helping allele causes all alleles associated with the helping allele to be (increasingly) indirectly selected (genetic hitchhiking). Significantly, as explained above, this includes rare *Neutral* alleles. Provided that partner search (α) is not too low, the stabilising force brought about by genetic hitchhiking is enough to give rare *Neutral* tags an overall advantage over common *Neutral* tags, allowing *Neutral* diversity to accumulate (Supplementary Figure 4c).

The long-term consequence of this linkage disequilibrium for the evolution of kin recognition depends upon the form of host-parasite coevolution. One outcome arises if parasites adapt relatively rapidly and have intermediate virulence (low *lag* & int. *d*), such that host-parasite interactions alone can maintain maximal tag diversity at *Resist*. In this case, the tag diversity at *Neutral* builds up to approximately equal the tag diversity at *Resist*, meaning maximal tag diversity is maintained at both loci. The population will be indifferent with regards to which candidate recognition locus is used, and so the long-term evolution is neutral with

regard to whether recognition is based upon *Resist* or *Neutral* (Supplementary Figure 2 area *vα*).

A second outcome arises if parasites adapt relatively slowly and have relatively high virulence (higher *lag* & *d*). In this case, host-parasite interactions will cause larger oscillations in the tags at the *Resist* locus. These oscillations make the *Resist* locus a less useful indicator of relatedness, compared to the *Neutral,* which is not subject to such oscillations, and so can have a higher tag diversity. Consequently, the population ultimately evolves towards using *Neutral* for kin recognition (Supplementary Figure 2 area *vβ*).

A third outcome arises if parasites have low virulence (low *d*). In this area of parameter space, recall that, if the parasite resistance locus (*Resist*) is exclusively used for kin recognition (fixed recognition locus; *Scenario 2* in Supplementary Table 1), the negative frequency dependent selection arising from host-parasite interactions is weaker (thanks to a low *d*) than the positive frequency dependent selection arising from social interactions, with the result that *Resist* diversity is lost. However, we find that, in this case, where a proportion of the population are using *Neutral* rather than *Resist* for kin recognition (*Scenario 3* in Supplementary Table 1), the positive frequency dependent selection at *Resist* arising from social interactions is reduced, because fewer individuals are using *Resist* for kin recognition. Importantly, positive frequency dependent selection at is reduced enough that diversity can accumulate and be maintained at *Resist*. This allows helping to be selected amongst *Resist*-choosing individuals, in turn allowing *Neutral* diversity to accumulate by hitchhiking. The result is that maximum diversity is maintained at *Resist* and *Neutral*, with the population ultimately indifferent with regards to which candidate recognition locus is used (Supplementary Figure 2 area *vγ*). It is notable that, in this area of parameter space, *Resist* diversity builds up when the recognition locus can evolve (*Scenario 3*), but not when the recognition locus is fixed at *Resist* (*Scenario 2*). The reason for this is that, when the recognition locus can evolve, *Resist* can evolve to be less involved in kin

recognition (phenotype uncoupling), which allows the negative frequency dependent selection arising from host-parasite interactions to exceed the positive frequency dependent selection arising from social interactions, stabilising *Resist* diversity.

Does letting the choice of recognition locus evolve increase the area of parameter space where genetic kin recognition is stable?

Overall, for extreme (low and high) partner search values (α), we found that: *Neutral* tag diversity builds up in approximately the same region of parameter space as it did when the recognition locus was fixed at *Neutral* (*Scenario 1*); *Resist* tag diversity builds up in approximately the same region of parameter space as it did when the recognition locus was fixed at *Resist* (*Scenario 2*) (Supplementary Figure 3).

However, when partner search was high but not maximal (intermediate α), we found that tag diversity can build up over a greater region of parameter space when the choice of recognition locus is allowed to evolve, by *genetic hitchhiking* (23–25) (Figure 5b / 6; Supplementary Figure 4c). In this area of parameter space, letting the choice of recognition locus evolve increases the extent to which genetic kin recognition is stable.

Additional comments on the parameters chosen for Figure 1b in the main text.

Figure 1B in the main text plots the ‘probability of being recognised and helped’ against ‘tag frequency’. It is an illustrative figure, showing that there may be a positive correlation between these variables, with the upshot that common tags receive more help, and are therefore selected, leading to the loss of tag diversity (Crozier’s paradox). The point of the figure was to choose illustrative parameter values that lead to Crozier’s paradox. This is why we chose to set the social encounter parameter to zero (α=0), meaning individuals cannot have multiple social encounters for a given social interaction. The lack of multiple social encounters, in this case,

generates the positive correlation between tag frequency and the probability of being helped, simply because, without multiple social encounters, individuals with rare tags are unlikely to find a tag-matched individual to interact with (meaning there is little opportunity to receive help).

We also assumed the following parameter values when generating this figure: b=0.4, c=0.1 & θ=0.25. These parameter values mean that the following Hamilton's Rule condition is satisfied: $\theta b>c$, where θ gives the genetic relatedness that would arise for individuals that acquire social encounters without conditioning on a tag (random encounters). We showed in Appendix 2 that satisfaction of this condition means that indiscriminate helping will be favoured, meaning the conditional helping allele is positively selected. We also assumed that there is no trait mutation (μ_{Trait}=0). The positive selection of the conditional helping allele in combination with the lack of trait mutation means that no defectors (cheaters) will persist in the population in the evolutionary long term (there is no mutation, and therefore no "mutation-selection balance").

A lack of cheaters in the population was the scenario modelled by Crozier in his original statement of the paradox (19). However, later work has shown that Crozier's paradox can still sometimes arise even in the presence of cheaters (2, 17, 18, 26). Our decision to assume the "no cheater" scenario for Figure 1b was therefore not motivated by an attempt to use parameter values that lead to Crozier's paradox. Rather, it was motivated by a technical decision to obtain a scenario where, for each 'tag frequency', we can obtain a unique value for 'probability of being helped'. In general, 'probability of being helped' cannot be worked out for a given tag frequency, because 'probability of being helped' will be dynamically changing. Furthermore, in general, the frequency of tags themselves will be dynamically changing, meaning you can't just take an equilibrium value for 'probability of being helped', because you can't force a tag to evolve to a desired equilibrium frequency. It is only possible to obtain a unique 'probability

of being helped' value for each tag frequency in the case where there are no cheaters, because in this case, 'probability of being helped' is simply given by the probability of social interaction. The probability of social interaction (and therefore, in this case, the probability of being helped) is given mathematically, for the α=0 case, by $\theta + (1 - \theta)x_i$, where x_i is tag frequency. This was the function plotted in Figure 1b.

Appendix 5: A mathematical description of linkage disequilibrium, and how it stabilises genetic kin recognition.

We have previously explained how genetic kin recognition may arise as a consequence of various statistical associations between alleles (linkage disequilibria). In this appendix, we provide the mathematical justification for these explanations.

First, we derive equations for the association (linkage disequilibrium) between: (*i*) neutral tag frequency and helping; (*ii*) parasite resistance allele frequency and helping; (*iii*) neutral tag frequency and parasite resistance allele frequency. We also show how equations for other associations, such as that between a *Choice* allele and neutral tag frequency, are easily obtained with slight notational changes. Our derivations follow the methodology, outlined in Kirkpatrick et al. (27), for calculating linkage disequilibria in multi-locus population genetic models (17, 25, 28, 29). Technically, linkage disequilibrium is given by the covariance in allelic state of genes at a pair of loci, and readers uninterested in the explicit derivation of these covariances may jump straight to the results in Equations A21, A25 & A27.

After deriving expressions for these three linkage disequilibria, we explain how they contribute to stabilising tag diversity. Specifically, we explain the importance of linkage disequilibrium in the evolution of kin recognition mediated by: (*i*) partner search, (*ii*) host-parasite coevolution, and (*iii*) genetic hitchhiking.

<u>Derivation of linkage disequilibria.</u>

We characterise *trait deviation* as follows. Let an individual have a trait value of 0 if it has the helping allele, and a trait value of 1 if it has the defection allele. The population average trait value is then given by $\bar{p}$, where $\bar{p}$ is the population average frequency of the helping allele

$\left(\bar{p} = \sum_{i=1}^{L_{max}} \sum_{m=1}^{L_{max}} \sum_{y=0}^{1} x_{i1my}\right)$. A given individual will therefore deviate in its trait value from the population average trait value. For individuals with the helping allele, this deviation will be $1 - \bar{p}$, and for individuals with the defection allele, this deviation will be $-\bar{p}$. The average trait deviation amongst individuals bearing a given neutral tag *i* will then be given by:

$$p_i - \bar{p}, \tag{A18}$$

where p_i is the frequency of the helping allele amongst individuals who have the neutral tag *i* $\left(p_i = \sum_{m=1}^{L_{max}} \sum_{y=0}^{1} x_{i1my}\right)$. The average trait deviation amongst the whole population is trivially given by zero $\left(\sum_{i=1}^{L_{max}} x_i(p_i - \bar{p}) = 0\right)$.

We characterise *neutral tag frequency deviation* as follows. Let each individual have a neutral tag frequency value that is (trivially) given by the population frequency of its neutral tag $\left(x_i = \sum_{j=0}^{1} \sum_{m=1}^{L_{max}} \sum_{y=0}^{1} x_{ijmy}\right)$. A given individual may therefore deviate in its neutral tag frequency value from the population average neutral tag frequency value. For individuals with the neutral tag *i*, this deviation will be:

$$x_i - \sum_{i=1}^{L_{max}} {x_i}^2, \tag{A19}$$

where the $\sum_{i=1}^{L_{max}} {x_i}^2$ term gives the population average neutral tag frequency (i.e. this will be one if there is only one neutral tag at fixation in the population). The average neutral tag frequency deviation amongst the whole population is trivially given by zero $\left(\sum_{i=1}^{L_{max}} x_i\left(x_i - \sum_{i=1}^{L_{max}} {x_i}^2\right) = 0\right)$.

For a given individual with a neutral tag *i*, the product of deviations over the *Trait* and *Neutral* loci is obtained by multiplying expressions A18 & A19 to give:

$$\left(x_i - \sum_{i=1}^{L_{max}} {x_i}^2\right)(p_i - \bar{p}). \qquad (A20)$$

If this multiplicatively combined joint deviation is positive, it means either that: (*i*) the individual has a more-common-than-average neutral tag and a greater-than-average expectation (based on its neutral tag identity) of being a helper; (*ii*) the individual has a less-common-than-average neutral tag and a lower-than-average expectation (based on its neutral tag identity) of being a helper. Conversely, if this multiplicatively combined joint deviation is negative, it means either that: (*i*) the individual has a more-common-than-average neutral tag and a lower-than-average expectation (based on its neutral tag identity) of being a helper; (*ii*) the individual has a less-common-than-average neutral tag and a greater-than-average expectation (based on its neutral tag identity) of being a helper.

The expected product of deviations over the *Trait* and *Neutral* loci, measured across all individuals in the population, is then obtained by averaging expression A20 over all neutral tags ({1,2,…,L_{max}}), to give:

$$D_{\{Trait,Neutral\}} = \sum_{i=1}^{L_{max}} x_i\left(x_i - \sum_{i=1}^{L_{max}} {x_i}^2\right)(p_i - \bar{p}). \qquad (A21)$$

Equation A21 gives the association (linkage disequilibrium) between neutral tag frequency and helping. It is a population-wide statistic, insofar that it refers to a characteristic of the population as a whole, rather than to a specific individual in the population. Technically, it is equal to the covariance in the allelic state of genes at the *Trait* and *Neutral* loci. A positive

value of $D_{Trait,Neutral}$ indicates that common neutral tags are associated with (technically: "covary with") the helping allele. Conversely, a negative value of $D_{Trait,Neutral}$ indicates that rare neutral tags are associated with (technically: "covary with") the helping allele. In Supplementary Figure 4, we plot $-D_{\{Trait,Neutral\}}$ rather than $D_{\{Trait,Neutral\}}$, as the former represents the association between *rare* neutral tags and helping.

We characterise *parasite resistance allele frequency deviation* as follows. Let each individual have a parasite resistance allele frequency value that is (trivially) given by the population frequency of its parasite resistance allele $\left(\tilde{x}_m = \sum_{i=1}^{L_{max}} \sum_{j=0}^{1} \sum_{y=0}^{1} x_{ijmy}\right)$. A given individual may therefore deviate in its parasite resistance allele frequency value from the population average parasite resistance allele frequency value. For individuals with the parasite resistance allele *m*, this deviation (in parasite resistance allele frequency value) will be:

$$\tilde{x}_m - \sum_{i=m}^{L_{max}} \tilde{x}_m{}^2 . \qquad (A22)$$

It is also convenient, at this point, to write down the deviation in parasite resistance allele frequency value amongst individuals with the neutral tag *i.* We will make use of this expression later:

$$\sum_{i=m}^{L_{max}} \frac{x_{im}\tilde{x}_m}{x_i} - \sum_{i=m}^{L_{max}} \tilde{x}_m{}^2 . \qquad (A23)$$

x_{im} is the population frequency of all individuals who have allele *i* at the *Neutral* locus as well as allele *m* at the *Resist* locus $\left(x_{im} = \sum_{j=0}^{1} \sum_{y=0}^{1} x_{ijmy}\right)$. The average parasite resistance allele

frequency deviation amongst the whole population is trivially given by zero $\left(\sum_{m=1}^{L_{max}} \tilde{x}_m\left(\tilde{x}_m - \sum_{i=m}^{L_{max}} \tilde{x}_m{}^2\right) = 0\right)$.

For a given individual with a parasite resistance allele m, the product of deviations over the *Trait* and *Resist* loci is obtained by multiplying expressions A18 & A22 to give:

$$\left(\tilde{x}_m - \sum_{i=m}^{L_{max}} \tilde{x}_m{}^2\right)(\tilde{p}_m - \bar{p}). \qquad (A24)$$

The expected product of deviations over the *Trait* and *Resist* loci, measured across all individuals in the population, is then is then obtained by averaging expression A24 over all parasite resistance alleles ($\{1,2,\ldots,L_{max}\}$), to give:

$$D_{\{Trait,Resist\}} = \sum_{m=1}^{L_{max}} \tilde{x}_m\left(\tilde{x}_m - \sum_{i=m}^{L_{max}} \tilde{x}_m{}^2\right)(\tilde{p}_m - \bar{p})\,. \qquad (A25)$$

Equation A25 gives the association (linkage disequilibrium) between tag frequency and helping. A positive value of $D_{\{Trait,Resist\}}$ indicates that common parasite resistance alleles are associated with (technically: "covary with") the helping allele. Conversely, a negative value of $D_{Trait,Resist}$ indicates that rare parasite resistance alleles are associated with (technically: "covary with") the helping allele. In Supplementary Figure 4, we plot $-D_{\{Trait,Resist\}}$ rather than $D_{\{Trait,Resist\}}$, as the former represents the association between *rare* neutral tags and helping.

For a given individual with a neutral tag i, the product of deviations over the neutral tag and parasite resistance loci is obtained by multiplying expressions A20 & A23 to give:

$$\left(x_i - \sum_{i=1}^{L_{max}} {x_i}^2\right)\left(\sum_{i=m}^{L_{max}} \frac{x_{im}\tilde{x}_m}{x_i} - \sum_{i=m}^{L_{max}} {\tilde{x}_m}^2\right). \qquad (A26)$$

The expected product of deviations over the *Neutral* and *Resist* loci, measured across all individuals in the population, is then is then obtained by averaging expression A26 over all neutral tags ({1,2,…,L_{max}}), to give:

$$D_{\{Neutral,Resist\}} = \sum_{i=1}^{L_{max}} x_i \left(\sum_{i=1}^{L_{max}} {x_i}^2 - x_i\right)\left(\sum_{i=m}^{L_{max}} {\tilde{x}_m}^2 - \sum_{i=m}^{L_{max}} \frac{x_{im}\tilde{x}_m}{x_i}\right). \qquad (A27)$$

Equation A27 gives the association (linkage disequilibrium) between neutral tag frequency and parasite resistance allele frequency. A positive value of $D_{Neutral,Resist}$ indicates that common neutral tags are associated with (technically: "covary with") common parasite resistance alleles. Conversely, a negative value of $D_{\{Neutral,Resist\}}$ indicates that rare neutral tags are associated with (technically: "covary with") common parasite resistance alleles, and vice versa.

We note that Equations A21 and A25 give associations between tag frequency (measured either at *Neutral* or *Resist*) and an allele segregating at a biallelic locus (in these cases, the conditional helping allele). It is easy to see how these equations can be modified to give the association between tag frequency and an allele segregating at a different biallelic locus, such as the *Choice* locus ($D_{\{Choice,Neutral\}}$, $D_{\{Choice,Resist\}}$). For instance, the p_i in Equation A21, which gives the proportion of individuals bearing tag *i* that have the conditional helping allele, can be changed to a variable denoting the proportion of individuals bearing tag *i* that have the *Resist*-choosing *Choice* allele. Similarly, the $\bar{p}$ in Equation A21, which gives the population frequency of helping, can be changed to a variable denoting the population

frequency of the *Resist*-choosing *Choice* allele. These modifications lead to an expression for $D_{\{Choice,Neutral\}}$.

Linkage disequilibrium and the three routes to genetic kin recognition.

The linkage disequilibrium equations (A21, A25, A27) are plotted for illustrative trials in Supplementary Figure 4. More generally, we find that there are three ways in which genetic kin recognition may evolve in our model. Linkage disequilibrium is important in two of these three cases.

Firstly, when kin discrimination is favoured ($R_{tag}\, b - c > 0$ is satisfied; Appendix 2), linkage disequilibrium builds up between rare *Neutral* tags and helping (i.e., $D_{\{Trait,Neutral\}}$ decreases). Under conditions of high partner search (α), this build-up of $-D_{\{Trait,Neutral\}}$ gives rare *Neutral* tags an overall advantage over common *Neutral* tags, allowing tag diversity to be maintained, facilitating kin recognition (Supplementary Figure 4a). Linkage disequilibrium between rare *Neutral* tags and helping drives the evolution of partner search-mediated kin recognition (2).

Secondly, when kin discrimination is favoured ($R_{tag}\, b - c > 0$ is satisfied; Appendix 2), and host-parasite coevolution maintains tag diversity at *Resist* (low *lag* & int. *d*), individuals will be able to use *Resist* to socially interact with their relatives, favouring helping (kin selection). Linkage disequilibrium between rare *Resist* alleles and helping ($-D_{\{Resist,Neutral\}}$) arises only transiently, and dissipates once maximal *Resist* diversity has been generated by host-parasite coevolution. The dissipation of $-D_{\{Resist,Neutral\}}$ occurs simply because, once all *Resist* tags have converged on the same population frequency, there can be no association between *Resist* tag frequency and helping, by definition! The helping allele continues to spread even after $-D_{\{Resist,Neutral\}}$ has dissipated, owing to standard kin selection (Supplementary Figure 4b). Linkage disequilibrium therefore does not drive the evolution of parasite-mediated kin

recognition. However, there are cases where host-parasite coevolution does not stabilise *Resist* diversity on its own (low d, or high d & *lag*), but a high partner search (α) can contribute to give rare *Resist* tags an overall advantage. In such cases, $-D_{\{Resist,Neutral\}}$ does build up and contribute to the stabilisation of kin recognition. However, this is simply partner search-mediated kin recognition, as described in the previous paragraph, acting on a parasite resistance locus rather than an otherwise-neutral recognition locus.

Thirdly, as stated in the previous paragraph, when kin discrimination is favoured ($R_{tag}\ b - c > 0$ is satisfied; Appendix 2), and host-parasite coevolution maintains tag diversity at *Resist* (*lag* low & d not too high), helping will be favoured amongst individuals who are using *Resist* to recognise kin (kin selection). This can open a third route to kin recognition if partner search (α) is intermediate. Linkage disequilibrium between rare *Neutral* tags and helping ($-D_{\{Trait,Neutral\}}$) builds up, owing to a co-selection of helping and rare *Neutral* tags amongst individuals that are using the *Neutral* allele to recognise kin. Individuals will evolve towards using *Resist* rather than *Neutral* for kin recognition, because there is more diversity at *Resist*, but as long as there is some *Choice* mutation (μ_{Choice}), some individuals will continue to use *Neutral*, which allows the linkage disequilibrium ($-D_{\{Trait,Neutral\}}$) to persist. The selection of helping (amongst *Resist*-using individuals) therefore indirectly selects for all alleles associated with helping, including rare *Neutral* alleles (Supplementary Figure 4c). Linkage disequilibrium between rare *Neutral* tags and helping drives the evolution of genetic hitchhiking-mediated kin recognition. Most other types of linkage disequilibrium, such as between rare *Resist* tags and helping ($-D_{\{Trait,Resist\}}$), and between rare *Resist* tags and rare *Neutral* tags ($D_{\{Resist,Neutral\}}$), arise only transiently (dissipating once *Resist* tags have equalised in frequency), meaning they cannot be long term drivers of genetic hitchhiking-mediated kin recognition (Appendix 7). Readers interested in how we know genetic hitchhiking is causing the build-up of *Neutral* tag diversity in this case, rather than, say, epistatic selection, should consult Appendix 7.

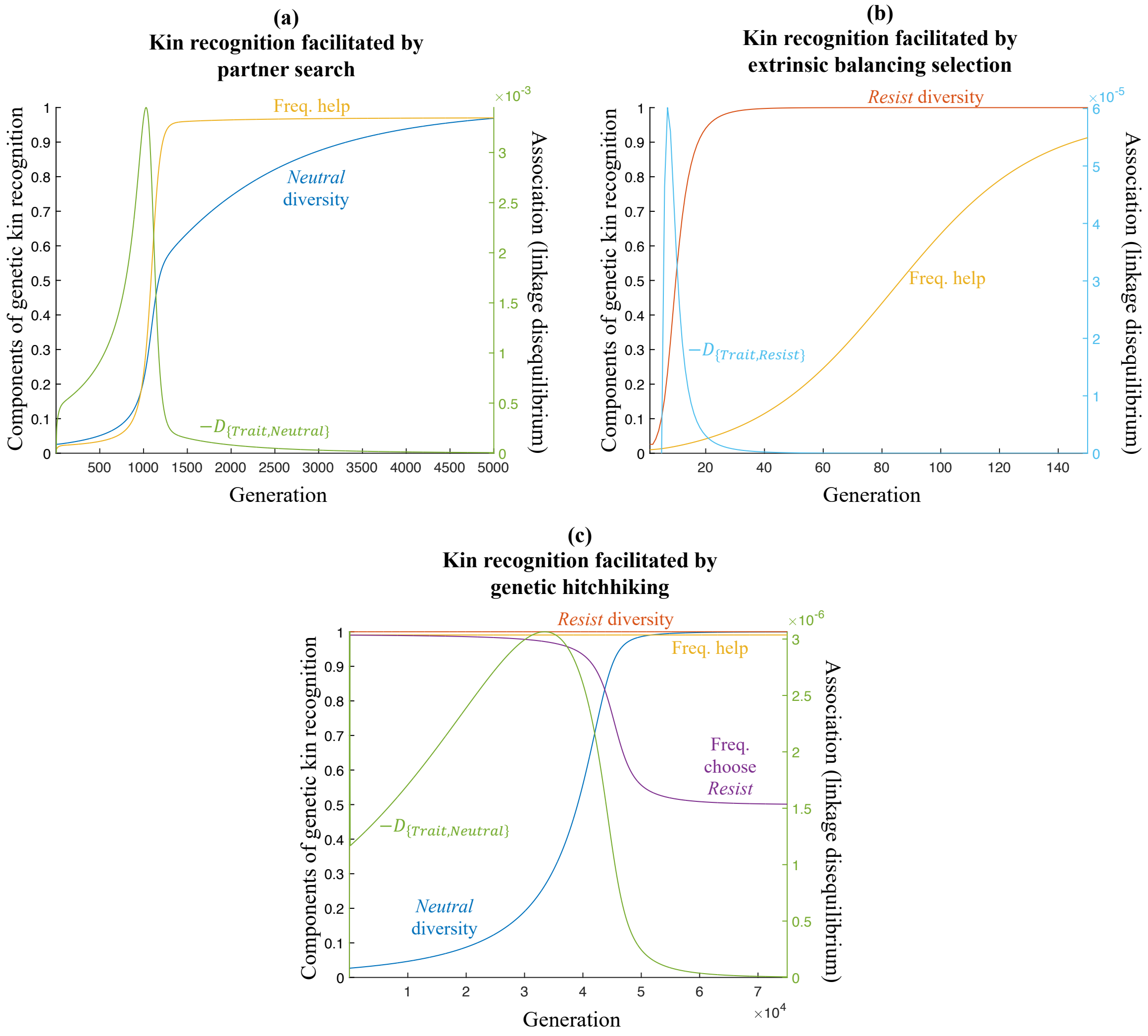

Supplementary Figure 4. Linkage disequilibrium and the routes to genetic kin recognition. The three panels show the three different ways in which genetic kin recognition (tag diversity + helping) may evolve in our model. Each panel represents a single illustrative trial, taken from a region of parameter space where kin discrimination is favoured by kin selection ($R_{tag}\ b > c$). (**A**) Partner search is high (α=1). Linkage disequilibrium between rare *Neutral* tags and helping ($-D_{\{Trait,Neutral\}}$) facilitates kin recognition, and persists until maximal tag diversity is acquired. (**B**) Host-parasite coevolution maintains tag diversity at *Resist* (*d*=0.6, *lag*=0, α=0). This allows individuals to interact with their relatives, favouring helping (kin selection). Linkage disequilibrium between rare *Resist* alleles and helping ($-D_{\{Resist,Neutral\}}$) arises only transiently, meaning it is not the long term driver of kin recognition. (**C**) Partner search is intermediate, and host-parasite coevolution maintains tag diversity

at *Resist* (d=0.6, lag=0, α=0.99). This allows individuals to interact with their relatives, favouring helping (kin selection). Linkage disequilibrium between rare *Neutral* tags and helping ($-D_{\{Trait,Neutral\}}$) builds up, favouring rare *Neutral* tags because of their association with helping (genetic hitchhiking). Linkage disequilibrium ($-D_{\{Trait,Neutral\}}$) persists until maximal *Neutral* diversity is acquired. Most other types of linkage disequilibrium, such as between rare *Resist* tags and helping ($-D_{\{Resist,Neutral\}}$) and between rare *Resist* tags and rare *Neutral* tags ($D_{\{Resist,Neutral\}}$), arise only transiently, meaning they cannot be long term drivers of kin recognition (Appendix 7). We assumed: b=0.3, c=0.1, μ_{Trait}=0.001, μ_{Choice}=0.001, L_{max}=10, (**A**) θ=0.08, (**B–C**) θ=0.25.

Appendix 6: Effect of mutation.

We have previously stated that some small amount of *Trait* and *Choice* mutation may sometimes be required for genetic kin recognition to be maintained. In this appendix, we explain why this is the case.

First, we describe why *Trait* mutation is often important, both in the simple context of *Scenario 1* (recognition locus fixed at *Neutral*), and in the more general context of *Scenario 3* (evolving recognition locus). Second, we describe why *Choice* mutation is often important.

Trait mutation

First, we consider the case where genetic kin recognition can only be based on a locus that is neutral aside from its role in kin recognition (i.e., all individuals are forced to use the *Neutral* recognition locus). Further results for this case were given in the analysis of *Scenario* 1. For tag diversity to build up at an endogenously evolving recognition locus (*Neutral*), *Trait* mutation (μ_{Trait}) sometimes needs to be above some non-zero threshold. Note that this threshold is generally very low, meaning the mutation requirement will tend to be met in natural populations. *Trait* mutation is important for the following reason.

At an endogenously evolving recognition locus (*Neutral*), kin selection causes defectors to spread amongst individuals bearing common tags (low relatedness), and conditional altruists to spread amongst individuals bearing rare tags (high relatedness; Figure 4). If the conditional altruism allele spreads (via rare-tag groups) more quickly than it is removed (via common-tag groups), which is often but not always the case when partner search (α) is maximal, the conditional altruism allele will increase in population frequency alongside rare tags, leading to *Neutral* tag equilibration (genetic kin recognition) and conditional altruism.

However, if the conditional altruism allele spreads (via rare-tag groups) more slowly than it is removed (via common-tag groups), the conditional altruism allele will decrease in population frequency. In this case, in the absence of *Trait* mutation (μ_{Trait}=0), the frequency of the conditional altruism allele will fall to approximately zero (fixation of the defection allele), resulting in defection. This may even occur in regions of parameter space where kin discrimination is favoured by kin selection (i.e., Equation A16 is satisfied) and partner search (α) is high. In other words, even if conditional altruism is favoured when all tags are at equal frequency (Equation A16 satisfied), the quick purging of conditional altruists amongst individuals bearing common tags may mean that tag frequencies do not successfully equilibrate, and defection evolves as a result.

However, if the conditional altruism allele spreads (via rare-tag groups) more slowly than it is removed (via common-tag groups), *but there is mutation at the trait locus*, the frequency of the conditional altruism allele will not fall all the way to zero. Rather, it will only fall as low as mutation-selection balance (non-zero). This means that, even if conditional altruists are being removed (via common tags) faster than they are being added (by rare tag groups), a baseline proportion of altruists will persist in the population due to the net mutation of defectors into altruists. Furthermore, the altruists that persist will disproportionately bear rare tags (linkage disequilibrium).

In this way, *Trait* mutation ensures that fitness differences between *Neutral* tags persist despite the potentially fast purging of conditional altruists bearing common *Neutral* tags. The persistence of *Neutral* tag fitness differences allows rare *Neutral* tags to increase in frequency alongside the conditional altruism allele. The common *Neutral* tags decrease in frequency, eventually reaching low enough population frequencies that the conditional helping allele is selected amongst individuals bearing these tags. After this point, the conditional helping allele is universally favoured (i.e. selected amongst individuals bearing all tags), resulting ultimately

in tag equilibration and the spread of the conditional helping allele to near-fixation (mutation-selection balance). It is for this reason that some *Trait* mutation (μ_{Trait}) is sometimes required for genetic kin recognition based on an otherwise-neutral recognition locus (*Neutral*) to evolve.

Second, we consider the case where the recognition locus used for genetic kin recognition can evolve to change, between either a locus that is involved in parasite resistance or a locus that is otherwise-neutral (i.e., individuals can use either *Neutral* or *Resist*). If partner search (α) is high, *Trait* mutation can influence whether tag diversity is maintained at *Resist* and / or *Neutral*, for the same reason that was given above for the case where individuals are forced to use an endogenously evolving recognition locus (*Neutral*). Essentially, when partner search (α) is high, rare tags can gain an advantage over common tags because they are less likely to be cheated in social interactions, but for this advantage to persist in the evolutionary long term, cheaters must not be completely purged from the population, and a small amount of *Trait* mutation guarantees this. Conversely, when partner search (α) is low, this advantage for rare tags (reduced likelihood of being cheated in social interactions) is negligible, meaning *Trait* mutation has a negligible effect on whether tag diversity is maintained at *Resist* and / or *Neutral.*

Choice mutation

In addition, *Choice* mutation can influence whether tag diversity can be maintained at *Neutral* by hitchhiking. Specifically, for tag diversity to build up at *Neutral* by genetic hitchhiking, *Choice* mutation (μ_{Choice}) sometimes needs to be above some non-zero threshold. Note again that this threshold is generally very low, meaning the mutation requirement will tend to be met in natural populations.

To recap, hitchhiking occurs when partner search (α) is intermediate, and parasites are rapidly adapting with intermediate or high virulence (low *lag* & int. / high *d*). In this region of

parameter space, host-parasite coevolution maintains tag diversity at *Resist*, but partner search (α) alone is insufficient to maintain tag diversity at *Neutral*. Helping is favoured amongst individuals who are using *Resist* to recognise kin (kin selection). Linkage disequilibrium between rare *Neutral* tags and helping ($-D_{\{Trait,Neutral\}}$) builds up amongst individuals who are using *Neutral* to recognise kin. Individuals will evolve towards using *Resist* to recognise kin, because there is more tag diversity at *Resist* than *Neutral*. This gives an overall advantage for the helping allele over the defection allele (positive selection). However, the linkage disequilibrium between *Neutral* tags and helping ($-D_{\{Trait,Neutral\}}$) will persist if enough individuals in the population continue to use *Neutral*. The positive selection of helping therefore leads to the indirect selection of rare *Neutral* alleles by genetic hitchhiking, allowing *Neutral* tag diversity to accumulate.

A non-zero threshold rate of *Choice* mutation ($\mu_{Choice}>0$) is sometimes required for hitchhiking to occur, for the following reason. Given that tag diversity builds up initially at *Resist*, and only subsequently at *Neutral*, there will initially be more tag diversity at *Resist* than *Neutral*. This means that *Resist* is more effective than *Neutral* in identifying genealogical kin, and as a result, the population initially evolves towards using *Resist* for kin recognition. If this evolution towards using *Resist* occurs rapidly relative to the rate at which *Neutral* tag diversity accumulates by hitchhiking, the population will evolve to the point where all *Choice* diversity is lost, meaning all individuals are using *Resist* for kin recognition. As the population approaches this state, the strength of selection acting on *Neutral* dissipates and, if this population state is reached, falls to zero. The reason why the strength of selection on *Neutral* dissipates is simply that, if all *Choice* diversity is lost and no-one is using *Neutral* for kin recognition, there can no longer be any co-selection of rare *Neutral* tags and conditional altruism. The consequence of this reduced selection on *Neutral* is that recombination causes

Neutral tags to lose their association with conditional helping. This prevents rare *Neutral* tags from being selected via an association with a positively selected allele (helping).

This scenario is less likely to occur if there is some *Choice* mutation (μ_{Choice}). This is because, if there is *Choice* mutation, it will never be the case that all *Choice* diversity is lost completely (loss of polymorphism). Rather, in the evolutionary long term, some individuals will necessarily be using the *Neutral* locus for kin recognition, because mutational pressure (mutation-selection balance) prevents the *Neutral*-choosing allele from being completely lost from the population. This means that the co-selection of rare *Neutral* tags and conditional altruism is never completely eradicated, which allows the association between *Neutral* tags and helping to persist in the long term. It is for this reason that some *Choice* mutation (μ_{Choice}) is sometimes required for tag diversity at a neutral recognition locus to accumulate by hitchhiking.

Appendix 7: Evidence for genetic hitchhiking.

We found that, when parasites adapt rapidly and with intermediate or high virulence (low *lag*, int. / high *d*), and when the social encounter parameter (α) is intermediate, tag diversity builds up at the *Neutral* locus by hitchhiking on a conditional helping allele that is under positive selection. The low *lag*, int. / high *d* requirement arises because this is what allows tag diversity to accumulate at the *Resist* locus, which is in turn what allows the conditional helping allele to experience overall positive selection. The int. α requirement arises because: if α is too high, neutral tag diversity will accumulate on its own, without needing to hitchhike; if α is too low, neutral tag diversity will never be able to accumulate, as the selective forces favouring common neutral tags are too strong to be overcome by indirect selection (hitchhiking). In this appendix, we explain how we know that hitchhiking on the conditional helping allele, rather than other forms of hitchhiking or epistatic selection, is what is responsible for stabilising neutral tag diversity in the area of parameter space characterised by low *lag*, int. / high *d*, int. α.

A preliminary point is that, in this area of parameter space (low *lag*, int. / high *d*, int. α), we are not claiming that hitchhiking is solely responsible (and *sufficient*) for stabilising neutral tag diversity. We are claiming that it is partially responsible (and *necessary*) for stabilising neutral tag diversity, and works alongside the standard, well documented epistatic co-selection of rare neutral tags and helping (2, 15–17, 26). Under high values of the social encounter parameter (α), this epistatic selection is sufficient for stabilising neutral tag diversity. However, in the area of parameter space that we are currently focused on (notably int. rather than high α), epistatic selection is insufficient, as can be seen by running a two-locus *Neutral–Trait* version of the model in this area of parameter space, and observing that neutral tag diversity does not accumulate. This is how we know that another selective force, in addition to the standard epistatic co-selection of rare neutral tags and helping, is stabilising neutral tag diversity in the full four-locus model. We will explain how we know that this additional

selective force is the hitchhiking of rare neutral tags on a positively selected conditional helping allele.

<u>An aside, on the use of allele-selection equations for analysing multi-locus models.</u>

Firstly, we note that one approach to identifying hitchhiking, which seems promising, would be to derive an equation for selection on neutral tags. The neutral tag selection equation could then be analysed to infer selective forces acting on the neutral tags. For instance, if the equation were to have a component that changes with the frequency of the allele, then this may imply the presence of frequency-dependent selection. A component that changes with linkage disequilibrium may imply the presence of hitchhiking. A component that changes with the identity of an allele at a different locus may imply epistatic selection. The neutral tag selection equation could be derived using the Price equation, or more simply by taking genotype fitness (given in Equations A1–3) and averaging it over all genotypic environments that a given neutral tag finds itself in (25, 27, 30).

Unfortunately, we are not able to take this exact approach, because the resulting neutral tag selection equation is too complex. That is, it features complex interactions between many selective processes, meaning we cannot use it to unambiguously say, for instance, "hitchhiking is driving this particular aspect of evolutionary change", because "hitchhiking" components of selection are weighted by many other selective processes, acting concurrently. Another reason we are not able to take this exact approach is that selection is contingent on allele frequencies and linkage disequilibria, which are constantly changing. It is unclear which allele frequency and linkage disequilibrium values to concentrate on when trying to draw general evolutionary principles from the neutral tag selection equation. One possible solution to these problems would be to simplify the neutral tag selection equation by making assumptions like quasi-

linkage equilibrium, but we cannot do that here, because most of the interesting evolutionary dynamics arise as a consequence of the *evolution* of linkage disequilibrium.

These problems mean that we have to take a slightly different approach. However, we will still derive and make use of a simplified neutral tag selection equation, which is accurate for the two locus (*Neutral–Trait*) version of the model.

<u>Possible reasons why neutral tag diversity is stabilised under low *lag*, int. / high *d*, int. α.</u>

First, we list all the possible ways in which neutral tag diversity might be being stabilised when moving from the two-locus *Neutral–Trait* model (where neutral tag diversity is not stable) to the full four-locus model (where neutral tag diversity is stable). We will then eliminate all but one of these possibilities, to isolate the selective force stabilising neutral tag diversity under low *lag*, int. / high *d*, int. α. The move from the two-locus to the four-locus model may:

1. Increase the linkage disequilibrium between rare neutral tags and helping, which means that rare neutral tags find themselves sharing a genotype with the conditional helping allele more often, which may increase the overall (epistatic) selection experienced by rare neutral tags, such that they are positively selected.
2. Increase the frequency of the helping allele, which in turn may generate increased (epistatic) selection on rare neutral tags, such that they are positively selected.
3. Allow rare neutral tags to experience a new component of positive selection, which is epistatic with *Resist* alleles, such that rare neutral tags are positively selected.
4. Allow rare neutral tags to enter linkage disequilibrium with *Resist* alleles under positive selection, and then spread by hitchhiking.
5. Allow rare neutral tags to experience a new component of positive selection, which is epistatic with a *Choice* allele, such that rare neutral tags are positively selected.

6. Allow rare neutral tags to enter linkage disequilibrium with a *Choice* allele under positive selection, and then spread by hitchhiking.
7. Allow rare neutral tags to spread by hitchhiking on a positively selected helping allele, which it is in linkage disequilibrium with.

Possibilities 1 & 2 refer to epistatic co-selection of rare neutral tags with the helping allele. As we mentioned above, this selective force (epistatic co-selection) is present even in the two-locus *Neutral–Trait* model, and is well documented (2, 15–17, 26). However, Possibilities 1 & 2 hypothesise that the strength of this selective force (epistatic co-selection) is increased in the four-locus model, relative to the two-locus *Neutral–Trait* scenario, and this is what allows rare neutral tags to be stabilised in the full model. In other words, the hypothesis is that having the opportunity to use a parasite resistance locus for kin recognition (somehow) leads to increased epistatic co-selection of rare neutral tags and helping. At this stage, our goal is to not give a plausible account of how having the opportunity to use a parasite resistance locus for kin recognition might increase epistatic selection, but rather to state it as a possibility that needs to be examined and potentially ruled out.

Possibilities 3–6 refer to selective forces arising through interactions of neutral tags with the "new" *Choice* and *Resist* loci, which are present in the four-locus but not the two-locus *Neutral–Trait* model. Possibility 7 refers to a type of hitchhiking that exploits the association between rare neutral tags and the conditional helping allele that is already there (i.e., this association builds up even in the two-locus *Neutral–Trait* model, as a consequence of the "well documented" epistatic co-selection of rare neutral tags and helping that we have mentioned several times). Together, these possibilities exhaust all the ways in which the neutral tags could be gaining their new advantage that leads to their overall positive selection. We now proceed to eliminate all but one of these possibilities. We reiterate that this "process of elimination" analysis is undertaken specifically in the region of parameter space characterised

by low *lag*, int. / high *d*, int. α – the results given in this appendix should not necessarily be understood as holding outside of this region of parameter space.

Ruling out Possibility 1

Possibility 1 is easily ruled out, because we find that linkage disequilibrium between rare neutral tags and helping is actually *reduced* in the four-locus model relative to the two-locus *Neutral–Trait* model (Supplementary Figure 5b). Therefore, rare neutral tags are *less* likely to share a genotype with the helping allele in the four-locus model, which reduces epistatic selection on them. It is easy to understand why linkage disequilibrium between rare neutral tags and helping is reduced in the full model – it is simply because, in the full model, neutral tags are not always used for kin recognition (sometimes the parasite resistance locus is used), which means they are less regularly co-selected alongside helping, which reduces the rate at which linkage disequilibrium builds up by selection (2, 15–17, 26).

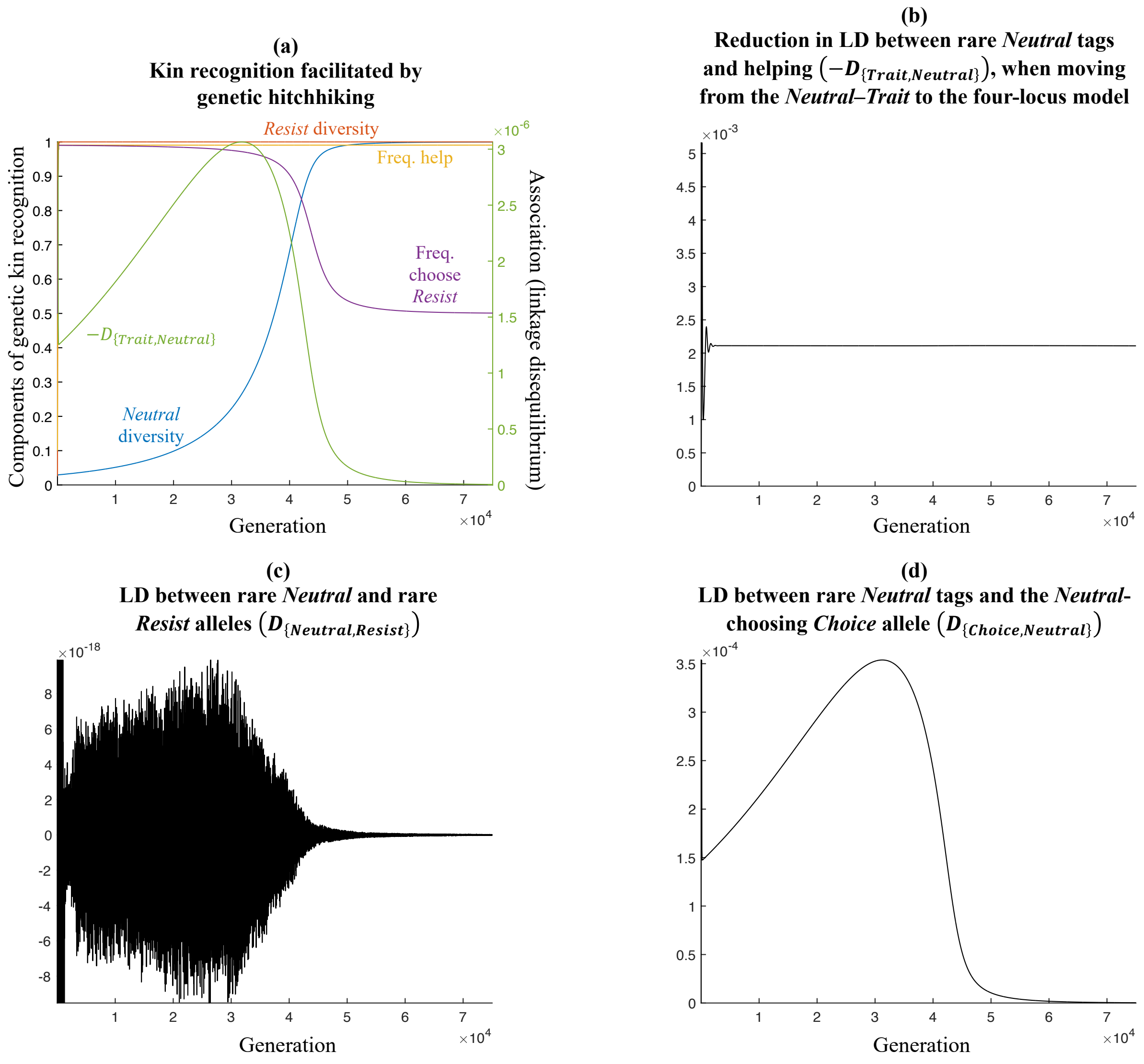

Supplementary Figure 5. Components of linkage disequilibrium when neutral tag diversity is stabilised by hitchhiking on a positively selected helping allele. Data is plotted for a single illustrative trial, in which partner search is intermediate, and host-parasite coevolution maintains tag diversity at *Resist* (d=0.6, lag=20, α=0.99, b=0.3, c=0.1, μ_{Trait}=0.001, μ_{Choice}=0.001, L_{max}=10, θ=0.25). (**A**) This allows individuals to interact with their relatives, favouring helping (kin selection). Linkage disequilibrium between rare *Neutral* tags and helping ($-D_{\{Trait,Neutral\}}$) builds up, favouring rare *Neutral* tags because of their association with helping (genetic hitchhiking). Linkage disequilibrium ($-D_{\{Trait,Neutral\}}$) persists until maximal *Neutral* diversity is acquired. (**B**) Linkage disequilibrium between rare *Neutral* tags and helping ($-D_{\{Trait,Neutral\}}$) decreases when moving from the two-locus *Neutral–Trait* model to the four-locus model. (**C**) Linkage disequilibrium between rare *Resist* tags and rare *Neutral*

tags ($D_{\{Resist,Neutral\}}$) is approximately zero (note that the y-axis scale is very small, and that this is a plot of a line graph – the rapid fluctuations give the illusion of a shaded area). (**D**) Linkage disequilibrium between rare *Neutral* tags and the *Neutral*-choosing *Choice* allele ($D_{\{Choice,Neutral\}}$) builds up.

Ruling out Possibility 2

Possibility 2 is partly true. That is, the frequency of the helping allele does in fact increase when moving from the two-locus *Neutral–Trait* model to the four-locus model. The reason for this increase is that, in the two-locus *Neutral–Trait* model, the helping allele experiences overall negative selection (its positive epistasis with rare neutral tags is too weak), but in the four-locus model, it experiences overall positive selection, because it can use *Resist* diversity to engage in precise kin recognition and socially interact with relatives, which favours helping by kin selection. However, the second part of Possibility 2 is that this increase in helping frequency in turn increases (epistatic) selection on rare neutral tags. We find that this is not the case, and we rule out Possibility 2 on this basis.

To show that increased helping frequency does not lead to increased selection on rare neutral tags, we derive and analyse an allele fitness equation, of the type that we discussed in the subsection above titled "An aside, on the use of allele-selection equations for analysing multi-locus models". However, for our present purposes, we only need to derive an allele fitness equation for the two-locus *Neutral–Trait* model, which is simple enough that we can analyse it. Specifically, by analysing the *Neutral–Trait* allele fitness equation, we can prove that an increase in the frequency of the helping allele, accompanied by a nonpositive change in the linkage disequilibrium between rare neutral tags and helping, cannot increase the fitness of rare tags, meaning rare tags do not experience increased selection. Given that, when ruling out Possibility 1, we showed that there is a decrease in the linkage disequilibrium between rare neutral tags and helping, this means that the increase in helping frequency observed in the full model is not the reason why neutral tag diversity is being stabilised in this model.

We provide the mathematical proof that that an increase in the frequency of the helping allele, accompanied by a nonpositive change in the linkage disequilibrium between rare neutral tags and helping, does not increase the fitness of rare tags. The proof proceeds by: (*i*) deriving the fitness of a neutral tag *i* in the two-locus *Neutral–Trait* model (w_i); (*ii*) deriving the fitness of neutral tag *i* when there is a possible change in the population helper frequency accompanied by a possible change in the linkage disequilibrium between rare neutral tags and helping (w_i'); (*iii*) obtaining an expression for the *change* in tag fitness ($w_i' - w_i$), and showing that, when the change in population helping frequency is *positive* and the change in linkage disequilibrium between rare tags and helping is *nonpositive*, the fitness of a rare tag (*i.e.*, a tag for which $x_i < \sum_l^{L_{max}} x_l^2$) will never increase. Readers uninterested in the mathematical details of this proof may skip to the next subsection.

Equation A28 below gives the fitness of a neutral tag *i* in the two-locus *Neutral–Trait* model, which we denote by w_i. Technically, Equation A28 gives the average fitness of all individuals bearing the neutral tag *i*. It is obtained by simplifying the genotype fitness functions (Equations A1–3), so that only the selective forces present in the two-locus *Neutral–Trait* model remain, and then taking an average over all genotypic environments experienced by the neutral allele *i*. Note that, in Equation A28, the selective consequences of competition are written down explicitly, as opposed to being subsumed in some high-level parameter like the "*A*" parameter of Equations A1–3. Recall that x_i here denotes the population frequency of the neutral tag *i*, and p_i denotes the proportion of individuals bearing the neutral tag *i* that are helpers. Note also that the *l* subscript denotes a random tag segregating in the population, which may or may not be the focal tag *i*.

$$w_i = 1 + (b - c)\left(\frac{p_i(\theta + (1-\theta)x_i)}{1 - \alpha(1 - x_i)(1 - \theta)} - \sum_l^{L_{max}} x_l \frac{p_l(\theta + (1-\theta)x_l)}{1 - \alpha(1 - x_l)(1 - \theta)}\right). \qquad (A28)$$

It is instructive to re-write this with the notation $p_l = \zeta_l + \bar{p}$, where $\bar{p}$ is the population helper frequency, and ζ_l is the deviation of tag *l*'s helper proportion from the mean tag helper proportion (27). Note that ζ_l is constrained such that the helper proportion for a given tag *l* cannot go above one or below zero $(-\bar{p} \leq \zeta_l \leq 1 - \bar{p})$. Note also that the average helper proportion deviation, taken over all individuals in the population, is trivially given by zero $\left(\sum_l^{L_{max}} x_l \zeta_l = 0\right)$. We obtain:

$$w_i = 1 + (b - c)\left(\frac{(\zeta_i + \bar{p})(\theta + (1 - \theta)x_i)}{1 - \alpha(1 - x_i)(1 - \theta)} - \sum_l^{L_{max}} x_l \frac{(\zeta_l + \bar{p})(\theta + (1 - \theta)x_l)}{1 - \alpha(1 - x_l)(1 - \theta)}\right). \quad (A29)$$

We now ask how the fitness of neutral tag *i* is affected by a possible change in the overall population frequency of the conditional helping allele, accompanied by a possible change in *Neutral–Trait* linkage disequilibrium. Specifically, we assume that the population frequency of the helping allele is multiplied by the factor *z*. Note that *z* is constrained such that the new population helper frequency cannot go above one or below zero $\left(0 \leq z \leq {}^{1}/_{\bar{p}}\right)$. The new population helper frequency is therefore $\bar{p}' = z\bar{p}$. We capture the possible change in linkage disequilibrium by allowing the helper proportion of each tag to deviate from the mean tag helper proportion by a new amount. $\hat{\zeta}_l$ is the new deviation of tag *l*'s helper proportion from the mean tag helper proportion. Note that $\hat{\zeta}_l$ is constrained such that the helper proportion for a given tag *l* cannot go above one or below zero $(-z\bar{p} \leq \hat{\zeta}_l \leq 1 - z\bar{p})$. Note also that the average deviation is trivially given by zero $\left(\sum_l^{L_{max}} x_l \hat{\zeta}_l = 0\right)$.The new fitness of neutral tag *i*, which we denote by w_i', is:

$$w_i' = 1 + (b - c)\left(\frac{(\hat{\zeta}_i + z\bar{p})(\theta + (1-\theta)x_i)}{1 - \alpha(1 - x_i)(1-\theta)} - \sum_{l}^{L_{max}} x_l \frac{(\hat{\zeta}_l + z\bar{p})(\theta + (1-\theta)x_l)}{1 - \alpha(1 - x_l)(1-\theta)}\right). \quad (A30)$$

We can use Equations A29 & A30 to obtain the change in fitness experienced by tag *i* as a consequence of the change in population helper frequency (*z*) and linkage disequilibrium ($\hat{\zeta}_l$ *cf.* ζ_l). This change in tag fitness is obtained by subtracting the right-hand-side of Equation A29 from the right-hand-side of Equation A30, leading to:

$$w_i' - w_i = \left(1 + (b - c)\left(\frac{(\hat{\zeta}_i + z\bar{p})(\theta + (1-\theta)x_i)}{1 - \alpha(1 - x_i)(1-\theta)} - \sum_{l}^{L_{max}} x_l \frac{(\hat{\zeta}_l + z\bar{p})(\theta + (1-\theta)x_l)}{1 - \alpha(1 - x_l)(1-\theta)}\right)\right)$$

$$- \left(1 + (b - c)\left(\frac{(\zeta_i + \bar{p})(\theta + (1-\theta)x_i)}{1 - \alpha(1 - x_i)(1-\theta)} - \sum_{l}^{L_{max}} x_l \frac{(\zeta_l + \bar{p})(\theta + (1-\theta)x_l)}{1 - \alpha(1 - x_l)(1-\theta)}\right)\right). \quad (A31)$$

We now proceed to simplify Equation A31, first by cancelling out the 1s, then factoring out the *b–c* terms and removing internal brackets:

$$w_i' - w_i = (b-c)\left(\frac{\left(\hat{\zeta}_i + z\bar{p}\right)(\theta + (1-\theta)x_i)}{1-\alpha(1-x_i)(1-\theta)} - \sum_l^{L_{max}} x_l \frac{\left(\hat{\zeta}_l + z\bar{p}\right)(\theta + (1-\theta)x_l)}{1-\alpha(1-x_l)(1-\theta)}\right.$$

$$\left. - \frac{(\zeta_i + \bar{p})(\theta + (1-\theta)x_i)}{1-\alpha(1-x_i)(1-\theta)} + \sum_l^{L_{max}} x_l \frac{(\zeta_l + \bar{p})(\theta + (1-\theta)x_l)}{1-\alpha(1-x_l)(1-\theta)}\right). \qquad (A32)$$

We simplify again by factorising:

$$w_i' - w_i = (b-c)\left(\frac{\left(\bar{p}(z-1) + \hat{\zeta}_i - \zeta_i\right)(\theta + (1-\theta)x_i)}{1-\alpha(1-x_i)(1-\theta)}\right.$$

$$\left. - \sum_l^{L_{max}} x_l \frac{\left(\bar{p}(z-1) + \hat{\zeta}_l - \zeta_l\right)(\theta + (1-\theta)x_l)}{1-\alpha(1-x_l)(1-\theta)}\right). \qquad (A33)$$

Equation A33 gives the change in fitness experienced by a neutral tag *i* when there is a change in the population helper frequency (*z*) accompanied by a change in linkage disequilibrium ($\hat{\zeta}_l$ *cf.* ζ_l).

We can use Equation A33 to examine whether rare tags can gain a selective advantage as a consequence of the population helper frequency increasing alongside a nonpositive change in the linkage disequilibrium between rare tags and helping. To do so, we assume that: *z*>1 (population helper frequency has increased); $\begin{cases} \hat{\zeta}_i, \zeta_i < 0, & for\ x_i > \sum_l^{L_{max}} x_l^2 \\ \hat{\zeta}_i, \zeta_i > 0, & for\ x_i < \sum_l^{L_{max}} x_l^2 \end{cases}$ (rare tags are in linkage disequilibrium with helping); $\left|\hat{\zeta}_l\right| \leq |\zeta_l|$ (the linkage disequilibrium between rare tags and helping has decreased). With these restrictions on the possible values for $z, \hat{\zeta}_i$ & ζ_i, we find using Equation A33 that: $\begin{cases} w_i' - w_i \geq 0, & for\ x_i > \sum_l^{L_{max}} x_l^2 \\ w_i' - w_i = 0, & for\ x_i = \sum_l^{L_{max}} x_l^2 \\ w_i' - w_i \leq 0, & for\ x_i < \sum_l^{L_{max}} x_l^2 \end{cases}$. This proves that selection on a

rare tag (*i.e.*, a tag for which $x_i < \sum_l^{L_{max}} x_l^2$) can never increase as a consequence of the population helper frequency increasing alongside a nonpositive change in the linkage disequilibrium between rare tags and helping. We can therefore rule out Possibility 2.

<u>Ruling out Possibilities 3 & 4.</u>

Possibilities 3 & 4 are easily ruled out, because we find that linkage disequilibrium does not accumulate between neutral tags and *Resist* alleles (Appendix 5; Supplementary Figure 5c). This means that rare neutral tags cannot experience epistatic co-selection with *Resist* alleles (which would lead to an observable increase in linkage disequilibrium between them), nor can they hitchhike with positively selected *Resist* alleles (which would require linkage disequilibrium between them). The absence of linkage disequilibrium between neutral tags and *Resist* alleles therefore indicates that neither epistatic nor hitchhiking interactions are taking place between them.

<u>Ruling out Possibilities 5 & 6.</u>

In the full model, linkage disequilibrium builds up between rare neutral tags and the *Neutral*-choosing *Choice* allele (Supplementary Figure 5d). The reason for this is simply that, amongst individuals using the *Neutral* locus for kin recognition (i.e., amongst *Neutral*-choosing individuals), rarer neutral tags have a direct selective advantage over common neutral tags, because they facilitate more precise kin recognition. However, amongst individuals using the *Resist* locus for kin recognition (i.e., amongst *Resist*-choosing individuals), there is no direct selection on neutral tags. This direct selection of rare neutral tags amongst *Neutral*-choosing individuals, but not *Resist*-choosing individuals, is a form of epistatic selection, and leads to the accumulation of linkage disequilibrium between rare neutral tags and the *Neutral*-choosing *Choice* allele.

However, this component of selection, which is epistatic in the four-locus model, is still present in the two-locus *Neutral–Trait* model – it is the selective consequence of using neutral tags to engage in kin discrimination. In the full model, this component of selection only arises for individuals bearing the *Neutral*-choosing allele, but in the two-locus *Neutral–Trait* model, all individuals are forced to use the *Neutral* locus for kin recognition, meaning this component of selection arises for everyone. The upshot is that epistatic co-selection with a directly-selected *Neutral*-choosing allele cannot be the cause of *Neutral*-diversity build-up in the four-locus model, because it does not lead to any new component of selection on rare tags, that didn't already exist in the two-locus *Neutral–Trait* model. We can therefore rule out Possibility 5.

A further consequence of this component of selection already existing in the two-locus *Neutral–Trait* model is that the rare neutral tags cannot be spreading by hitchhiking on the *Neutral*-choosing *Choice* allele in the four-locus model. This is because, although there is linkage disequilibrium between rare neutral tags and the *Neutral*-choosing *Choice* allele, and the *Neutral*-choosing *Choice* allele is (at least transiently) positively selected, there is no new component of direct selection on the *Neutral*-choosing *Choice* allele that the rare neutral tags can "hitchhike on" in the four-locus model, because they are already fully exposed to this component of selection in the two-locus *Neutral–Trait* model. We can therefore rule out Possibility 6.

<u>Settling on Possibility 7</u>

This leaves us with Possibility 7, which is the only explanation from the exhaustive list of possibilities that cannot be ruled out. Possibility 7 therefore provides the explanation for why neutral tag diversity is stabilised under conditions of low *lag*, int. / high *d*, int. α. We have arrived at this conclusion through a process of elimination. In addition to this, there is *positive* evidence consistent with Possibility 7, such as the observation that linkage disequilibrium

between rare neutral tags and helping persists for a long period of time in the four-locus model, and only dissipates once neutral tag diversity has stabilised (Supplementary Figure 4). This positive evidence was given in Appendix 5.

Appendix 8: Additional Discussion.

Why should there be pleiotropy between parasite resistance and genetic kin recognition?

Why one should there be pleiotropy between these seemingly unrelated traits. The main reason to expect pleiotropy, given by advocates of the hypothesis that host-parasite coevolution stabilises genetic kin recognition, is as follows (17, 19–21). Firstly, we would expect individuals to want to base kin recognition on the locus in the genome that has the most allelic diversity, because this allows the most precise kin recognition. Secondly, we would expect parasite resistance loci to be good candidates for being the most genetically diverse loci in the genome. Therefore, we would expect the pleiotropy to evolve, to facilitate precise kin recognition.

This argument is stronger than simply invoking pleiotropy between unrelated traits, because there is a principled reason to expect the pleiotropy to evolve. However, we do not fully endorse the argument, for reasons given throughout the paper.

The importance of kinship (pedigree) relatedness.

Relatedness between social partners can be achieved in two main ways. The first is kinship (common ancestry). If two individuals share a common ancestor (e.g. grandmother), then they are likely to have inherited similar genes, and therefore be genetically similar (related). Field biologists can often infer kinship relatedness simply by observing an animal's family tree (pedigree).

The second way to achieve relatedness is by gene-matching. Kin-discriminating animals find their social partners by matching tags. A consequence of tag matching is that relatedness will be maximised (=1) at the tag locus. This is simply because any individuals that use tag-matching as a basis for social interaction will (trivially) be genetically identical at the locus responsible for encoding the tag. Furthermore, any loci that are in linkage disequilibrium

with the tag locus may have heightened relatedness. This includes loci that are physically linked to the tag locus (i.e. on the same chromosome), as well as other loci, like the *Trait* locus, that may be associated with the tag locus due to co-selection.

The key difference between kinship and gene-matching, as causes of relatedness, is that kinship increases genetic similarity (relatedness) at all loci, whereas gene-matching only increases genetic similarity at a subsection of loci within the genome (the tag locus and those in linkage disequilibrium with it). This leads to the question – what type of relatedness is important for the evolution of discriminating altruism? Does discriminating altruism evolve in accordance with kinship (pedigree) relatedness, or tag locus relatedness, or something else? The first thing to note here is that the evolution of *any* social trait will technically proceed in accordance with the relatedness measured at the locus (or loci) responsible for encoding the trait (3, 5). In the context of our model, evolution proceeds in accordance with relatedness at the *Trait* locus. That is, only in generations where $R_{tag} b > c$ is satisfied, where R_{tag} is measured at the *Trait* locus (Equation A12 / 1), will the conditional helping allele increase in frequency. As we said before, *Trait* locus relatedness may deviate from kinship (pedigree) relatedness due to tag-trait linkage disequilibrium.

This might seem to imply that knowledge about kinship (pedigree) relatedness is insufficient for determining the outcome of social evolution. However, one important result of our analyses here and in ref. (2) is that, when kin discrimination successfully evolves, tags tend to evolve to obtain approximately equal frequencies and cheater loads, meaning there is no linkage disequilibrium at equilibrium. This means that *Trait* locus relatedness converges on kinship (pedigree) relatedness in the evolutionary long term (2). Therefore, it is kinship (pedigree) relatedness that tends to matter when we are thinking about evolutionarily stable social traits, as predicted by Hamilton and Grafen (3–5, 15).

This means we can interpret R_{tag} (Equation A12 / 1) as a coefficient of kinship (pedigree) relatedness. This is good news for empiricists, who can approximate, for natural populations, the coefficient of relatedness that features in Equation A12 / 1, without having to know genetic details like linkage disequilibrium.

<u>The presence of squares in many equations.</u>

In our analyses, the population average (neutral) tag frequency at a given point in time is given by $\sum_{i=1}^{L_{max}} x_i{}^2$ (e.g., Equation A19). This may seem counterintuitive – shouldn't the average tag frequency be given by $1/L$, where L is the number of segregating tags? Here, we explain why the square is required, leading to $\sum_{i=1}^{L_{max}} x_i{}^2$ rather than $1/L$. First, we give an intuitive non-technical explanation, and secondly, we give a technical explanation.

Imagine there are L=10 tags at a locus. Taking $1/L$ would then imply that the average tag frequency is 0.1. But what if 1 of the 10 tags is at frequency 0.9999, and the remaining 9 tags have frequencies that are divided up between the remaining 0.0001? It would be unsatisfactory to say that 0.1 is the average tag frequency in this case. This is why we used the square in our measure of tag frequency, as it gives an *effective* tag frequency that incorporates information about the abundance of each tag.

Now for the technical reason. Technically, we modelled tag frequency as a trait wielded by individuals. "Average tag frequency" is, technically, an *average taken over individuals*. To take this average, the individual-level trait (given by its tag frequency) needs to be weighted by the proportion of individuals in the population exhibiting that particular trait (and this proportion is also given by the individual's tag frequency), and these values are then summed to obtain the average. The multiplication of tag frequency by tag frequency gives rise to the square.

Squares pop up in other equations, not just the average tag frequency expression. The presence of a square usually implies that the average tag frequency is relevant. We note that the presence of squares does not represent an arbitrary modelling decision, but rather, squares are needed to accurately: construct our selection equations, calculate linkage disequilibria, etc.

Supplementary References

1. E. H. Simpson, Measurement of Diversity. *Nature* **163**, 688–688 (1949).
2. T. W. Scott, A. Grafen, S. A. West, Multiple social encounters can eliminate Crozier's paradox and stabilise genetic kin recognition. *Nat Commun* **13**, 3902 (2022).
3. W. D. Hamilton, The genetical evolution of social behaviour. I. *J Theor Biol* **7**, 1–16 (1964).
4. A. Grafen, A geometric view of relatedness. *Oxford surveys in evolutionary biology* **262**, 391–397 (1985).
5. W. D. Hamilton, The genetical evolution of social behaviour. II. *J Theor Biol* **7**, 17–52 (1964).
6. D. C. Queller, Genetic relatedness in viscous populations. *Evol Ecol* **8**, 70–73 (1994).
7. P. D. Taylor, Altruism in viscous populations-an inclusive fitness model. *Evol Ecol* **6**, 352–356 (1992).
8. P. D. Taylor, Inclusive fitness in a homogeneous environment. *Proc R Soc Lond B Biol Sci* **249**, 299–302 (1992).
9. F. Rousset, *Genetic Structure and Selection in Subdivided Populations (MPB-40)* (Princeton University Press, 2004).
10. S. A. Frank, *Foundations of Social Evolution* (Princeton University Press, 1998).
11. G. S. Faria, A. Gardner, Does kin discrimination promote cooperation? *Biol Lett* **16**, 20190742 (2020).
12. G. S. Faria, A. Gardner, P. Carazo, Kin discrimination and demography modulate patterns of sexual conflict. *Nat Ecol Evol* **4**, 1141–1148 (2020).
13. D. C. Queller, Expanded social fitness and Hamilton's rule for kin, kith, and kind. *Proc Natl Acad Sci U S A* **108**, 10792–10799 (2011).
14. D. C. Queller, A general model for kin selection. *Evolution (N Y)* **46**, 376–380 (1992).

15. A. Grafen, Do animals really recognize kin? *Anim Behav* **39**, 42–54 (1990).

16. R. Axelrod, R. A. Hammond, A. Grafen, Altruism via kin-selection strategies that rely on arbitrary tags with which they coevolve. *Evolution (N Y)* **58**, 1833–1838 (2004).

17. F. Rousset, D. Roze, Constraints on the origin and maintenance of genetic kin recognition. *Evolution (N Y)* **61**, 2320–2330 (2007).

18. L. Holman, J. S. van Zweden, T. A. Linksvayer, P. d'Ettorre, Crozier's paradox revisited: maintenance of genetic recognition systems by disassortative mating. *BMC Evol Biol* **13**, 211 (2013).

19. R. H. Crozier, Genetic clonal recognition abilities in marine invertebrates must be maintained by selection for something else. *Evolution (N Y)* **40**, 1100–1101 (1986).

20. J. Field, C. Accleton, W. A. Foster, Crozier's Effect and the Acceptance of Intraspecific Brood Parasites. *Current Biology* **28**, 3267-3272.e3 (2018).

21. A. Gardner, S. A. West, Social Evolution: The Decline and Fall of Genetic Kin Recognition. *Current Biology* **17**, R810–R812 (2007).

22. S. C. Stearns, *The Evolution of Life Histories* (Oxford University Press, 1992).

23. N. H. Barton, Genetic hitchhiking. *Philos Trans R Soc Lond B Biol Sci* **355**, 1553–1562 (2000).

24. J. Maynard Smith, J. Haigh, The hitch-hiking effect of a favourable gene. *Genet Res* **23**, 23–35 (1974).

25. A. Gardner, S. A. West, N. H. Barton, The Relation between Multilocus Population Genetics and Social Evolution Theory. *Am Nat* **169**, 207–226 (2007).

26. V. A. A. Jansen, M. van Baalen, Altruism through beard chromodynamics. *Nature* **440**, 663–666 (2006).

27. M. Kirkpatrick, T. Johnson, N. Barton, General Models of Multilocus Evolution. *Genetics* **161**, 1727–1750 (2002).

28. D. Roze, F. Rousset, Multilocus models in the infinite island model of population structure. *Theor Popul Biol* **73**, 529–542 (2008).

29. N. H. Barton, M. Turelli, Natural and sexual selection on many loci. *Genetics* **127**, 229–255 (1991).

30. G. R. Price, Selection and Covariance. *Nature* **227**, 520–521 (1970).
